# The Role of Five-Membered Heterocycles in the Molecular Structure of Antibacterial Drugs Used in Therapy

**DOI:** 10.3390/pharmaceutics15112554

**Published:** 2023-10-29

**Authors:** Aura Rusu, Ioana-Maria Moga, Livia Uncu, Gabriel Hancu

**Affiliations:** 1 Pharmaceutical and Therapeutic Chemistry Department, Faculty of Pharmacy, George Emil Palade University of Medicine, Pharmacy, Science and Technology of Targu Mures, 540142 Targu Mures, Romania; moga.ioana-maria@stud18.umfst.ro (I.-M.M.); gabriel.hancu@umfst.ro (G.H.); 2 Scientific Center for Drug Research, “Nicolae Testemitanu” State University of Medicine and Pharmacy, 8 Bd. Stefan Cel Mare si Sfant 165, MD-2004 Chisinau, Moldova; livia.uncu@usmf.md

**Keywords:** heterocycles, five-membered heterocycles, antibiotics, antibacterials, nitrogen heterocycles, oxygen heterocycles, sulfur heterocycles, biological activity, drug design, drug discovery

## Abstract

Five-membered heterocycles are essential structural components in various antibacterial drugs; the physicochemical properties of a five-membered heterocycle can play a crucial role in determining the biological activity of an antibacterial drug. These properties can affect the drug’s activity spectrum, potency, and pharmacokinetic and toxicological properties. Using scientific databases, we identified and discussed the antibacterials used in therapy, containing five-membered heterocycles in their molecular structure. The identified five-membered heterocycles used in antibacterial design contain one to four heteroatoms (nitrogen, oxygen, and sulfur). Antibacterials containing five-membered heterocycles were discussed, highlighting the biological properties imprinted by the targeted heterocycle. In some antibacterials, heterocycles with five atoms are pharmacophores responsible for their specific antibacterial activity. As pharmacophores, these heterocycles help design new medicinal molecules, improving their potency and selectivity and comprehending the structure-activity relationship of antibiotics. Unfortunately, particular heterocycles can also affect the drug’s potential toxicity. The review extensively presents the most successful five-atom heterocycles used to design antibacterial essential medicines. Understanding and optimizing the intrinsic characteristics of a five-membered heterocycle can help the development of antibacterial drugs with improved activity, pharmacokinetic profile, and safety.

## 1. Introduction

Heterocyclic compounds play a significant role in sustaining life, as they are abundant in nature. The genetic material comprises crucial heterocycles such as purine and pyrimidine bases. Additionally, several heterocycles are structural components of common therapeutic drugs, either obtained by chemical synthesis or naturally occurring [1,2]. The significance of heterocycles in drug design stems from their ability to modify the drug candidate’s physicochemical characteristics, biological impacts, pharmacokinetics, and toxicological profile [2]. Numerous recent studies have targeted therapeutic agents the structures of which are based on different heterocycles [3,4,5,6]. In the last decade, scientists have successfully synthesized numerous heterocyclic compounds to develop new antibacterials capable of treating infections caused by drug-resistant bacterial strains. Certain five-membered heterocycles containing two or three heteroatoms (e.g. thiazole, benzothiazole, thiazolidinone, triazole, and others) are crucial structural components in various antibacterials [7].

The physicochemical properties imprinted by a five-membered heterocycle are associated with the biological activity of the antibacterial drug (spectrum of activity and potency) and its pharmacokinetic, pharmacologic, and toxicological profiles. Highlighting the relationships between certain five-membered heterocycles in the molecular structure of the antibacterial drugs and their biological properties can be the basis for many other rational design studies for discovering new antibiotics.

### 1.1. Antibacterials Shortage

In the ongoing fight against bacterial infections, there is an urgent need for new antibacterials. Antibiotic resistance appears inevitable, and pharmaceutical companies consistently demonstrate little interest in financing the research of new antibacterials [8]. New compounds from plants, animals, or bacteria are tested for their antimicrobial properties. Discovering and developing new antibacterials can be scientifically challenging [9].

The lack of antibiotics results from production problems, supply chain disruptions, increased demand, regulatory difficulties, and pharmaceutical companies discontinuing specific drugs. Major pharmaceutical corporations stopped developing antibacterials despite the ongoing need for novel antimicrobial medications. Developing antibacterials can be costly, and the return on investment may not be as attractive as other therapeutic areas. Due to the high costs of clinical trials, new regulatory uncertainty over approval requirements, and a low rate of return, companies have left aside the antibacterial research and development field. The lack of antibiotics might affect patient care. Potential outcomes include more extended hospital stays, a higher risk of complications, poor or delayed treatment for infections, and the development of antibiotic resistance. The antibiotic regulatory pathway is rigorous, which can increase the time and cost of bringing new drugs to market, discouraging pharmaceutical companies from pursuing antibiotic research. The Food and Drug Administration’s (FDA) dropping of approval of antibacterials is a reflection of pharmaceutical companies’ unwillingness to invest in antibacterial drug development [10,11].

Shortages can affect a wide range of antibiotics, both generic and branded. Addressing the issue of antibiotic resistance requires a multi-pronged approach involving governments, public health organizations, pharmaceutical companies, and the scientific community. Older antibiotics are scarce and have been taken off the market for known or unknown reasons. Many countries do not have access to many older, possibly helpful, and occasionally "forgotten" antibiotics since they were either never released or discontinued. Older antibiotics that have disappeared from use can be revived to minimize this burden [12,13]. The management of antibiotic shortages involves a team of healthcare experts. To promote proper antibiotic use, healthcare professionals must maintain effective patient communication, offer alternative treatments when necessary, and follow antimicrobial stewardship guidelines [14].

### 1.2. Bacterial Resistance Phenomenon

The rise of antibiotic resistance is a grave and urgent global health concern. The consequences of antibiotic-resistant infections are significant, leading to increased mortality, prolonged illness, higher healthcare costs, and limited treatment options. The gravest issue is the increased bacteria resistance to standard antibiotics, even to last-choice medications such as vancomycin. When these antibiotics become ineffective, limited treatment options remain, and infections can become virtually untreatable. The alarming increase in a problem that affects public health worldwide and necessitates international cooperation is confirmed by the speed with which resistance genes can spread worldwide. The World Health Organization (WHO) identified this phenomenon as a significant health issue in response to the worldwide observation of an alarming increase in the population of multi-drug-resistant strains [15,16]. Unfortunately, antibiotic resistance-related studies are becoming more prevalent every year. Antibiotic-resistant infections significantly burden healthcare systems, with more extended hospital stays, increased use of healthcare resources, and higher costs [17].

Antibiotic resistance has many negative effects: (a) failure to respond to treatment increases the length of illness and the number of deaths; (b) longer hospitalization and illnesses raise the possibility of spreading an infection to more persons in the community; (c) when a first-line antibiotic is no longer effective, the need to switch to second- or third-line antibiotics, which are always more expensive and occasionally more toxic; (d) first-line antibiotic resistance is more likely to develop in low-income countries due to the scarcity of numerous second- and third-line antibiotics; (e) the number of medications available in low-income countries to treat bacterial infections is declining, and the essential drug list does not include all the necessary antibiotics; (f) the advancements of modern medicine are under jeopardy due to antibiotic resistance; chemotherapy, organ transplants, and some procedures become more dangerous without the efficient antibiotics [8].

Fewer new antibiotics are being developed, and the pipeline for innovative antibacterial drugs remains relatively small.

### 1.3. FDA-Approved Antibiotics Whose Structure Includes Five-Members Heterocycles

Over time, many antibiotics that have been approved for use in therapy contain in their structure a heterocycle with five atoms or even more. In Table 1, we have chronologically summarized the antibiotics approved by the FDA in the last forty years and the essential heterocycles with five atoms in their molecular structure.

### 1.4. Aim of the Work

The five-membered heterocycles are among the most significant structural components of pharmaceuticals. They are used to optimize potency and selectivity through bioisosterism, pharmacokinetic, and toxicological features by providing numerous options to modify the antibacterials’ lipophilicity and solubility. Thus, in this review, we identified the majority of the antibacterial drugs used in therapy, which contain five-membered heterocycles, to better understand the properties that these heterocycles confer to an antibacterial agent. The antibiotics that include a particular five-membered heterocycle belong to structurally different classes with different mechanisms of action. The review addresses the pressing global health challenge of bacterial infections and antibiotic resistance by strategically incorporating five-membered heterocyclic rings into drug molecules. Consequently, our work aims to provide a well-structured collection of helpful information regarding antibacterials containing five-membered heterocycles for the rational design of new antibacterials. 

## 2. Materials and Methods

References were gathered from the Clarivate Analytics, ScienceDirect, PubMed, and Google Books databases, using as the primary keywords the terms “heterocycles”, “five-membered heterocycles”, “nitrogen heterocycles”, “oxygen heterocycles”, and “sulfur heterocycles”, combined with “antibiotics” or “antibacterials”. These keywords were also combined with the name of representative heterocycles, such as “pyrrolidine”, “pyrrole”, “furan”, and so on. Furthermore, other keywords such as “biological activity”, “drug design”, “drug discovery”, and “drug candidates” were combined with the specific name of the five-membered heterocycle. The references were chosen if they contained appropriate details concerning the primary topic of our review. The chemical structures were drawn with Biovia Draw 2022 (https://discover.3ds.com/biovia-draw-academic, accessed on 9 August 2023) [20]. IUPAC names of the compounds were used from the PubChem database (https://pubchem.ncbi.nlm.nih.gov/, accessed on 12 July 2023) [21].

## 3. Five-Membered Heterocycles Used in the Design of Antibacterial Drugs

Heterocyclic compounds contain at least two distinct atoms (either as ring atoms or as members of the ring) in the ring. The actual ring is referred to as a heterocycle. The total number of ring atoms and the type is crucial since it determines the ring size. Three-membered rings are the minor shape conceivable. The most significant rings in the antibiotic design are heterocycles, with five and six members [22]. 

The five-membered heterocycles used in antibacterial drug design contain one to four heteroatoms as follows:

- One heteroatom (nitrogen, oxygen, or sulfur);
- Two heteroatoms (oxygen and nitrogen; sulfur and nitrogen atoms);
- Three heteroatoms (three nitrogen atoms, e.g., triazoles, and one sulfur and two nitrogen atoms, e.g., thiadiazoles);
- Four heteroatoms (tetrazoles).

The most common heterocycles with five atoms in the molecular structure of approved antibiotics are presented in Table 2 and are individually addressed in the following sections.

## 4. Five-Membered Heterocycles Containing Nitrogen Atoms

Five-membered heterocycles are believed to originate from the cyclopentadienyl compound. They possess properties such as conjugated dienes or acyclic amines, but with a nitrogen atom replacing the “-CH=” group. The characteristics of these compounds are closely linked to the non-participatory electron pair of the heteroatom. They have a planar pentagonal structure, with the six π electrons distributed over the five sp2 hybridized atoms. Each carbon atom contributes one electron, and the heteroatom donates two electrons to the aromatic sextet, which confers the aromaticity of the heterocyclic system. The two non-participating electrons of the nitrogen will contribute to the aromatic sextet and are delocalized throughout the heterocycle [1]. These heterocycles are less susceptible to being deprotonated at the nitrogen or carbon atom through the action of nucleophiles. Weak nucleophiles will react with the cation produced by electrophiles, leading to addition or ring-opening reactions. The most reactive compound in this class in terms of compound reactivity is pyrrole. The resonance structures’ unevenly distributed energy causes greater reactivity [1]. 

According to an analysis conducted by Vitaku E. et al. (2014) on FDA-approved small compounds, N-heterocycles form the majority of the structural skeletons of pharmaceutical drugs on the market, accounting for about 84% of all molecules, and 59% of them contain at least one nitrogen heterocycle [23].

### 4.1. Pyrrolidine

Pyrrolidine is the first saturated family member of five-membered heterocycles with one nitrogen atom (Table 2). The heterocycle, commonly known as tetrahydropyrrole, is categorized as an aza cycloalkane (aza cyclopentane), a type of cyclic amine. The unsubstituted ones can participate in alkylation processes, while the substituted ones can be acylated or nitrosated. Pyrrolidine and N-substituted pyrrolidines go through reactions typical of secondary or tertiary alkylamines. Because they are cyclic amines, they can take part in these processes [22].

The compound is non-polar, flexible, and planar in structure. Its basicity is higher (pKa of 11.3) than that of the acyclic compound diethylamine (pKa of 10.49) [22,24]. Because the two alkyls “substituents” in the heterocycles, i.e., the ring carbons, are constrained back and away from the nitrogen lone pair, approach by an electrophile is made more accessible than in the case of diethylamine, where rotations of the C-N and C-C bonds interfere, pyrrolidine is a better nucleophile than diethylamine [25]. Both in natural and synthetic compounds, we can identify different modifications of pyrrolidines. The structure of several natural alkaloids, such as amathaspiramide A, B, and C, atropine, bgugaine and irniine, cocaine, codonopsinine and codonopsine, ficushispimine A and B, hygrine, nicotine, radicamine A and B, scalusamide A, and many more, have a pyrrolidine ring in their structure [24,26,27].

Five-membered heterocycles with nitrogen are employed more frequently as pharmacophores for various medicinal uses, free-standing rings, and spiro and polycyclic systems. Because of their capacity to interact with multiple essential enzymes, they have found particular use in pharmacotherapy for antibacterial or antiviral, antifungal, anticancer, and antidiabetic drugs [28]. As a pharmacophore group, pyrrolidine is found in the molecular structure of different classes of medicines, among them being an extensive series of antibiotics (Table 3) [29].

Pyrrolidine, as a structural element, is found in the molecule of some cephalosporins mentioned in Table 3, and its addition to their structure leads to numerous benefits. 

#### 4.1.1. Beta-Lactam Antibiotics

**Carbapenems**

**Figure 1:**
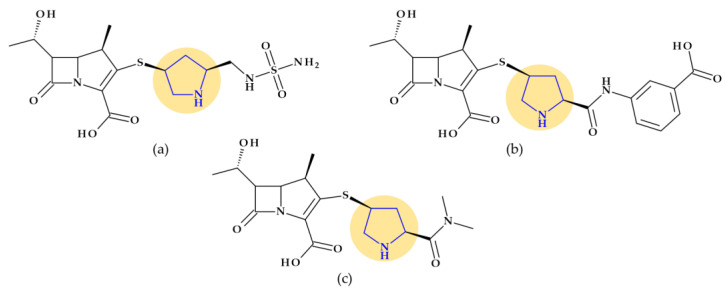
Chemical structure of carbapenems with the highlighted pyrrolidine nucleus: (**a**) doripenem, (**b**) ertapenem, and (**c**) meropenem.

Thienamycin, ((5*R*,6*S*)-3-(2-aminoethylsulfanyl)-6-[(1*R*)-1-hydroxyethyl]-7-oxo-1-azabicyclo [3.2.0]hept-2-ene-2-carboxylic acid), was the first carbapenem isolated from *Streptomyces cattleya*, which became the model substance for all carbapenems. Thienamycin’s chemical instability led to the development of similar compounds with improved stability. The first synthesized, more stable N-formimidoyl derivative was imipenem; several other optimized carbapenems have been developed lately [30,38]. Among the carbapenems that contain a pyrrolidine heterocycle in the molecular structure are doripenem, ertapenem, and meropenem (Figure 1). The pyrrolidine ring expanded the spectrum of activity and improved the potency and stability of these newly optimized compounds [30,39].

Some details related to the spectrum of antibacterial activity of the three compounds are presented below. Compared to currently available penicillins, cephalosporins, and beta-lactam/beta-lactamase inhibitor combinations, carbapenems exhibit an overall larger antibacterial spectrum in vitro. Doripenem is effective against Gram-positive bacteria. Thus, doripenem, ertapenem, and meropenem are slightly more effective against Gram-negative bacteria. When used against *Pseudomonas aeruginosa* and *Acinetobacter baumannii*, doripenem is more efficient than meropenem and is least susceptible to hydrolysis by carbapenemases. Meropenem is more efficient than ertapenem against *Pseudomonas aeruginosa*. Also, meropenem is efficient against multidrug-resistant *Mycobacterium tuberculosis* if combined with clavulanic acid (a beta-lactamase inhibitor) [30]. Targeting severe hospital-acquired infections with uncertain etiologies is a common usage of meropenem. However, carbapenems are commonly used as the last choice of antibiotics [39].

**Cephalosporins**

**Figure 2:**
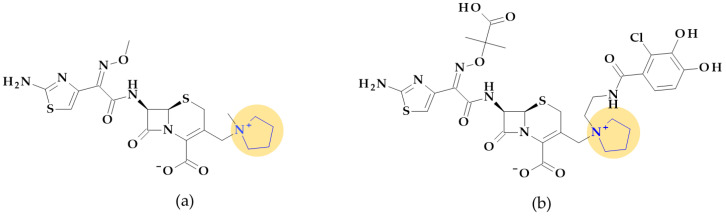
Chemical structure of cephalosporins with the highlighted pyrrolidine nucleus: (**a**) cefepime and (**b**) cefiderocol [31,42].

*Cefepime.* The first approved cephalosporin that has a pyrrolidine fragment in its structure is cefepime, ((6*R*,7*R*,*Z*)-7-(2-(2-aminothiazol-4-yl)-2-(methoxyimino)acetamido)- 3-((1-methylpyrrolidinium-1-yl)methyl)-8-oxo-5-thia-1-aza-bicyclo [4.2.0]oct-2-ene-2-carboxylate) (Figure 2a), a fourth-generation cephalosporin for parenteral use [31,40]. The activity spectrum of cefepime includes Gram-positive and Gram-negative bacteria. Cefepime is a parenteral cephalosporin used to treat infections with susceptible pathogens such as infections of the skin and soft tissues, complex intra-abdominal infections (associated with metronidazole), complicated and uncomplicated urinary tract infections (UTIs), pneumonia, and empirically neutropenic fever. The molecule has an amphoteric character and passes through the porins found in the cell walls of Gram-negative bacteria due to the methyl-pyrrolidine residue, where the nitrogen atom is quaternary. At the same time, their ability to penetrate cell wall porins can be explained based on the low lipophilicity conferred by the pyrrolidine moiety (log P of 0.46) [31,40,41].

*Cefiderocol*. Another cephalosporin that contains a pyrrolidine ring is cefiderocol, ((6*R*,7*R*)-7-[[(2Z)-2-(2-amino-1,3-thiazol-4-yl)-2-(2-carboxypropan-2-yloxyimino)acetyl]ami-no]-3-[[1-[2-[(2-chloro-3,4-dihydroxybenzoyl)amino]ethyl]pyrrolidin-1-ium-1-yl]methyl]-8-oxo-5-thia-1-azabicyclo [4.2.0]oct-2-ene-2-carboxylate) (Figure 2b). This fifth-generation cephalosporin is particularly effective against Gram-negative bacteria, including carbapenem-resistant bacteria. Cefiderocol is a mixture of catechol-type siderophores and cephalosporins from a structural standpoint. This compound is carried over the bacterial cell’s outer membrane and into the periplasm using specific iron transporter channels. Furthermore, cefiderocol has shown proven structural stability against hydrolysis by serine- and metallo-lactamases, including clinically significant carbapenemases [42,43]. The pyrrolidine nucleus confers increased antibacterial activity, increased stability of the nucleus against β-lactamases and an amphoteric character due to the quaternary nitrogen atom that increases the water solubility of the molecule [44].

With a minimum inhibitory concentration (MIC) of less than 4 mg/L for the majority of Enterobacteriaceae, *Pseudomonas aeruginosa*, and *Acinetobacter baumannii* isolates, cefiderocol exhibits high in vitro potency against pathogenic carbapenem-resistant Gram-negative bacteria [45] as a result of the high binding affinities for penicillin-binding proteins (PBP), especially PBP3 from *Escherichia coli*, *Klebsiella pneumoniae*, *Pseudomonas aeruginosa*, and *Acinetobacter baumannii* [46]. Cefiderocol’s practical application in the treatment of Gram-negative infections as part of the early access program (PERSEUS Study) has just undergone a retrospective analysis [47].

**Figure 3:**
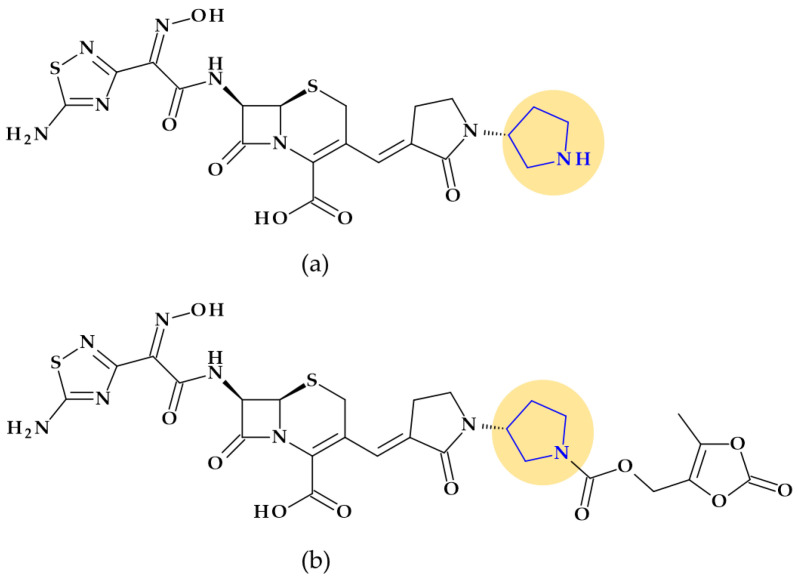
Chemical structure of (**a**) ceftobiprole and (**b**) ceftobiprole medocaril (prodrug), with the highlighted pyrrolidine nucleus [48].

*Ceftobiprole.* Pyrrolidine is also found in the molecular structure of ceftobiprole, ((6*R*,7*R*)-7-[[(2*Z*)-2-(5-amino-1,2,4-thiadiazol-3-ylidene)-2-nitroso-1-oxoethyl]amino]-8-oxo-3-[(E)-[2-oxo-1-[(3R)-3-pyrrolidinyl]-3-pyrrolidinylidene]methyl]-5-thia-1-azabicyclo [4.2.0]oct-2-ene-2-carboxylic acid), another fifth-generation cephalosporin (Figure 3). Because of its low solubility in water, ceftobiprole is administered as its water-soluble prodrug, ceftobiprole medocaril, which rapidly transforms into the active drug, diacetyl, and carbon dioxide; medocaril is the name of the fragment 5-methyl-2-oxo-1,3-dioxol-4-yl)methoxycarbonyl substituted at the nitrogen atom of the pyrrolidine heterocycle [48]. Due to its ability to inhibit abnormal PBP2a in methicillin-resistant *Staphylococcus aureus* (MRSA) and PBP2b and PBP2x in beta-lactam-resistant pneumococci, ceftobiprole is a unique parenteral extended-spectrum cephalosporin that is effective against both Gram-negative and Gram-positive bacteria that are resistant to antibiotics. Additionally, ceftobiprole is effective against *Pseudomonas aeruginosa* susceptible strains, AmpC overproducers, and Enterobacteriaceae that are not producing extended-spectrum beta-lactamases or carbapenemases [48,49,50].

The ceftobiprole molecule resembles the pentaglycine fragment due to the planarity of the pyrrolidine residue [50]. The MRSA’s high resistance to beta-lactam antibiotics is known to be facilitated by the expression of PBP2a, a penicillin-binding protein (PBP) [51]. Ceftobiprole interacts with the PBP2a and PBP2x to attach to their active sites, forming an antibiotic-acyl PBP2a complex. Between the residues of tyrosine Tyr446, methionine Met641, and threonine Thr600, respectively, is the pyrrolidine residue, which participates in a hydrogen bond with the sulfur atom of Met641. Pyrrolidine forms a π-π interaction with the tyrosine residue Tyr446 and hydrophobic interactions with the Thr600 threonine residue. The molecule’s planarity and hydrophobicity are crucial to access the active site of PBP2a enzymes. These types of interactions have been highlighted by molecular docking studies [50].

#### 4.1.2. Fluoroquinolones

**Figure 4:**
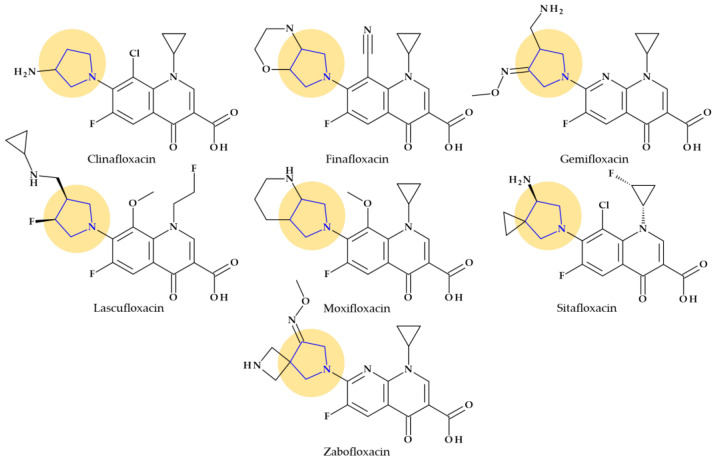
Chemical structures of fluoroquinolones with the highlighted pyrrolidine nucleus.

Fluoroquinolones constitute a different class of antibacterial significant for antibacterial therapy. The pyrrolidine moiety in position C7 is an essential structural component for some representatives such as clinafloxacin, finafloxacin, gemifloxacin, lascufloxacin, moxifloxacin, sitafloxacin, and zabofloxacin (Figure 4).

Among the benefits of pyrrolidine moiety is an increased spectrum of activity against Gram-positive bacteria, including MRSA, improved pharmacokinetic profile (increased half-life) and bioavailability [34,52,53]. However, the pyrrolidine substituent in the C7 position of fluoroquinolones has been associated by several authors with some of the side effects comprised in Table 4 [54].

The antibacterial fluoroquinolones that contain a pyrrolidine nucleus in their chemical structure are briefly presented below.

*Clinafloxacin.* Clinafloxacin (7-(3-aminopyrrolidin-1-yl)-8-chloro-1-cyclopropyl-6-fluoro-4-oxoquinoline-3-carboxy-lic acid) is a fourth-generation fluoroquinolone antibacterial agent. In the C7 position, clinafloxacin presents a 3-amino pyrrolidine substituent (Figure 4) [55]. Clinafloxacin proved efficient against most Gram-positive, Gram-negative, and anaerobic bacteria. Numerous studies have shown that clinafloxacin has a wide range of antibacterial activity, good tissue penetration and bioavailability, a prolonged serum half-life, enhanced safety and tolerability, and acceptable pharmacokinetics. However, clinafloxacin has several drawbacks, including poor solubility in its original form and insufficient stability in aqueous solution. Also, clinafloxacin was associated with severe side effects such as phototoxicity and the prevalence of hypoglycemia and, consequently, it was withdrawn from the market in 1999 [55,56,57].

*Finafloxacin.* Finafloxacin, 7-[(4a*S*,7a*S*)-3,4,4a,5,7,7a-hexahydro-2*H*-pyrrolo [3,4-b][1,4]oxazin-6-yl]-8-cyano-1-cyclopropyl-6-fluoro-4-oxoquinoline-3-carboxylic acid, has a chiral cyano-substituent, pyrrole-oxazine component, with a zwitterionic chemical structure (Figure 4) [58,59]. Under acidic environments, finafloxacin exhibits higher antibacterial activity. This property differentiates finafloxacin from other fluoroquinolones and is appropriate for specific infection sites, including the skin, soft tissues, vagina, and urinary tract. The highest level of bactericidal activity was found at pH 5–6. The approved indication as otic suspension of finafloxacin is acute otitis externa produced by *Pseudomonas aeruginosa* and *Staphylococcus aureus* [59].

*Gemifloxacin.* Gemifloxacin, 7-[(4*Z*)-3-(aminomethyl)-4-methoxyiminopyrrolidin-1-yl]-1-cyclopropyl-6-fluoro-4-oxo-1,8-naphthyridine-3-carboxylic acid, is a fourth-generation fluoroquinolone antibiotic [60], a 1,4-dihydro-1,8-naphthyridine derivative. The spectrum of activity is improved by heterocyclic substitution at C7, especially against Gram-negative bacteria. At the C7 position, gemifloxacin presents an unusual substituent: a methoxyiminopyrrolidine substituted with an aminomethyl fragment (at the pyrrolidine C3 position) (Figure 4) [31,61]. This fluoroquinolone was used orally to treat mild to moderate respiratory tract infections from susceptible microorganisms. However, gemifloxacin has been connected to a few cases of acute liver damage [60]. Though gemifloxacin had improved antibacterial action compared to moxifloxacin, it was withdrawn in 2009 by the producer due to adverse effects, primarily rash [61,62,63].

*Lascufloxacin.* The oral version of this lascufloxacin, 7-[(3*S*,4*S*)-3-[(cyclopropylamino)methyl]-4-fluoropyrrolidin-1-yl]-6-fluoro-1-(2-fluoroethyl)-8-methoxy-4-oxoquinoline-3-carboxylic acid, was licensed in Japan in 2019 to treat respiratory diseases, including community-acquired bacterial pneumonia (CABP) and ear, nose, and throat infections. An uncommon structural fragment can be found in the lascufloxacin chemical structure at position C7. It is about a primary pyrrolidine heterocycle 3-substituted with a (cyclopropyl amino)methyl moiety and 4-substituted with a fluorine atom (Figure 4). The interaction with DNA gyrase or topoisomerase IV requires this position. Previously, the representative of clinafloxacin also demonstrated that an amino pyrrolidine fragment enhances activity against Gram-positive pathogens. Lascufloxacin showed a strong affinity for phosphatidylserine, the primary surfactant in alveolar epithelial fluid and a component of human cell membranes. Compared to levofloxacin, garenoxacin, and moxifloxacin, lascufloxacin has better tissue penetration (head and neck infections). Also, lascufloxacin is shown to be quite effective when used against Gram-positive bacteria, including resistant strains [34,64].

*Moxifloxacin.* Moxifloxacin is a fourth-generation fluoroquinolone antibiotic [65]. Structurally, moxifloxacin, 7-[(4a*S*,7a*S*)-1,2,3,4,4a,5,7,7a-octahydropyrrolo [3,4-b]pyridin-6-yl]-1-cyclopropyl-6-fluoro-8-methoxy-4-oxoquinoline-3-carboxylic acid, is an 8-methoxy fluoroquinolone with a bulky moiety at the C7 position (Figure 4) [61,65]. This fragment is a fused bicycle of pyrrolidine and piperidine. Because of this C7-azabicyclo side chain, it is more challenging to efflux the moxifloxacin out of the bacterial cell [66], [67]. Moxifloxacin is characterized by a broad-spectrum activity against Gram-positive and Gram-negative bacteria and anaerobes. Compared to other fluoroquinolones from older generations, moxifloxacin has increased efficacy against Gram-positive organisms such as pneumococci and is very potent against anaerobes [65].

*Sitafloxacin.* Sitafloxacin, (7-[(7*S*)-7-amino-5-azaspiro [2.4]heptan-5-yl]-8-chloro-6-fluoro-1-[(1R,2S)-2-fluorocyclopropyl]-4-oxoquinoline-3-carboxylic acid, is a fourth-generation derivative, a chloro-fluoroquinolone that has received approval in Japan (2008) and Thailand (2012). It includes the [(*7S)*-7-amino-5-azaspiro [2.4]heptanyl fragment, a pyrrolidinyl fragment enclosed in a spiro substituent at the C7 position (Figure 4) [34]. Sitafloxacin could be considered a clinafloxacin analog, which underwent optimization at N1 and C7 positions. In vitro, sitafloxacin proved efficacy against Gram-positive, Gram-negative, anaerobic bacteria and atypical pathogens. Furthermore, it is effective against strains that are resistant to other fluoroquinolones and multi-drug-resistant bacteria [68,69,70].

*Zabofloxacin.* A brand-new broad-spectrum fluoroquinolone that can be orally administered is zabofloxacin, 1-cyclopropyl-6-fluoro-7-[(8*Z*)-8-methoxyimino-2,6-diazaspiro [3.4]octan-6-yl]-4-oxo-1,8-naphthyridine-3-carboxylic acid (Figure 4) [59]. This novel fluoroquinolone has similarities to the fourth-generation antibiotic gemifloxacin. Zabofloxacin contains an unusual heterocycle at the C7 position, a spiro substituent (2,6-diazaspiro [3.4]octan) substituted with an imino methoxy group; a pyrrolidinyl fragment is present in the structure of the spiro substituent of zabofloxacin [34]. Zabofloxacin has two different forms in development: hydrochloride and aspartate. The principal bacterial strains that zabofloxacin acts against include Gram-negative and Gram-positive respiratory pathogens (a broad spectrum against respiratory pathogens), particularly drug-resistant *Neisseria gonorrhoeae* and *Streptococcus pneumoniae*. According to its approved indications, zabofloxacin can be orally taken to treat acute bacterial chronic obstructive pulmonary disease exacerbations [34,59].

#### 4.1.3. Lincosamides (Lincomycin and Clindamycin)

**Figure 5:**
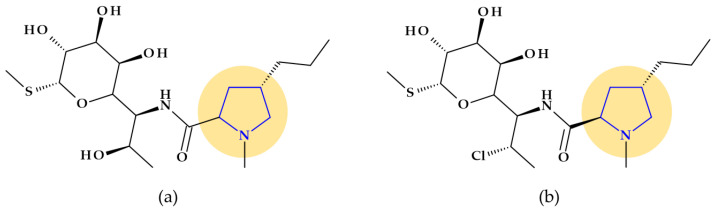
Chemical structure of lincosamides with the highlighted pyrrolidine nucleus: (**a**) lincomycin and (**b**) clindamycin.

Pyrrolidine is an essential pharmacophore group in the molecular structure of antibacterial lincosamides, lincomycin and clindamycin (Figure 5) [29]. Structurally, lincosamides consist of a thio-methylated carbohydrate unit joined by an amide bond to an N-methyl-pyrrolidine-carboxylic acid (proline) residue [31,71]. A fundamental function of pyrrolidine nitrogen is the formation of water-soluble salts with an apparent pKa of 7.6. Lincomycin, ((2*S*,4*R*)-N-[(1*R*,2*R*)-2-hydroxy-1-[(2*R*,3*R*,4*S*,5*R*,6*R*)-3,4,5-trihydroxy-6-(methylsulfanyl)oxan-2-yl]propyl]-1-methyl-4-propylpyrrolidine-2-carboxamide), is transformed into methyl α-thiolincosamide (the sugar moiety) and *trans*-L-4-n-propylhygric acid (the pyrrolidine moiety) when it is subjected to hydrazinolysis [29].

However, reports of severe diarrhea and the emergence of pseudomembranous colitis in patients receiving lincomycin (or clindamycin) have forced a reevaluation of the therapeutic use of these antibiotics [29]. Today, lincomycin is rarely used, replaced by its semisynthetic analog, clindamycin. Clindamycin, (2*S*,4*R*)-N-[(1*S*,2*S*)-2-chloro-1-[(2*R*,3*R*,4*S*,5*R*,6*R*)-3,4,5-trihydroxy-6-methylsulfanyloxan-2-yl]propyl]-1-methyl-4-propylpyrrolidine-2-carboxamide, is the only medication successfully used in clinical practice, although hundreds of lincomycin derivatives, including those created through total chemical synthesis, were obtained [71]. Also, pyrrolidine appears in the structure of clindamycin, a lincosamide with a broader spectrum of antibacterial activity and favorable pharmacokinetic profile versus lincomycin [29].

The timing and specificity of microbial protein synthesis stages are disrupted by lincosamides, which slow the growth or are fatal to the bacterium. Clindamycin’s three-dimensional structure closely resembles *L*-Pro-Met and the *D*-ribosyl ring of adenosine, which are found nearby at the 3′-ends of *L*-Pro-Met-tRNA and deacylated-tRNA for a brief period after the formation of a peptide bond between. This similarity could be explained by *L*-Pro-tRNA and *L*-Met-tRNA, which are involved in the molecular process through which clindamycin suppresses the synthesis of ribosomal proteins. The 3′ ends of *L*-Pro-Met-tRNA and deacylated-tRNA may, therefore, function as structural analogs of clindamycin and other lincosamides at the first stage of pre-translocation in the peptide elongation cycle [71].

#### 4.1.4. Streptogramins

*Quinupristin*/*Dalfopristin.* Antibiotics known as streptogramins are naturally produced by several species of the Streptomyces genus. The same bacterial species simultaneously produces the two subgroups of this class of antibiotics, type A and type B, at a ratio of roughly 70:30 [72,73]. Semi-synthetic water-soluble derivatives of pristinamycin IA (type B) and pristinamycin IIA (type A) were developed. Therefore, the formulation quinupristin-dalfopristin, marketed as Synercid^®^ (Pfizer, New York, NY, USA), exhibits activity against Gram-positive bacteria that are typically resistant to other medications, including MRSA and vancomycin-resistant *Enterococcus faecium*. The bacterial 50S ribosome is the primary target, and the formulation works by preventing the synthesis of bacterial proteins. In the bacterial ribosome, quinupristin and dalfopristin inhibit protein synthesis’s early and late stages. Both antibiotics contain a pyrrolidine heterocycle in their molecular structure (into a proline amino acid fragment) [36,74].

#### 4.1.5. Tetracyclines

Tetracyclines are recognized antibacterial agents with excellent broad-spectrum activity. They are effective against Gram-positive and Gram-negative bacteria and various spirochetes, *Mycoplasma*, *Rickettsiae*, and *Chlamydiae* [29,37].

**Figure 6:**
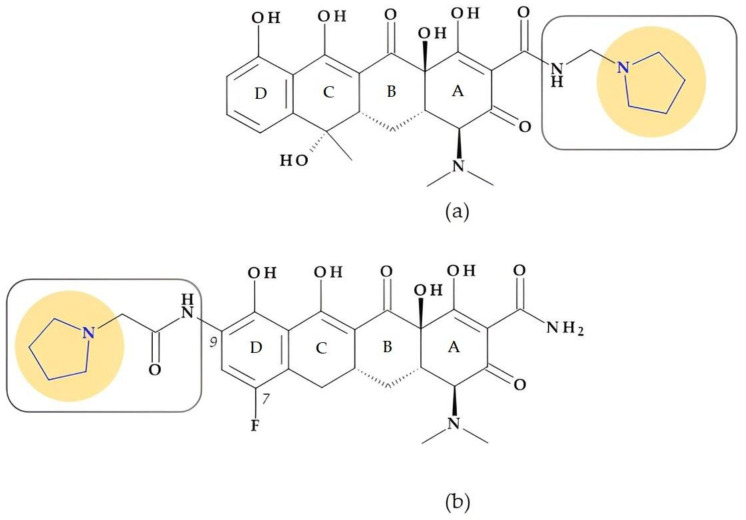
Chemical structure of tetracyclines with the highlighted pyrrolidine nucleus: (**a**) rolitetracycline and (**b**) eravacycline.

*Rolitetracycline.* N-(pyrrolidinomethyl)tetracycline or rolitetracycline, (4*S*,4a*S*,5a*S*,6*S*,12a*R*)-4-(dimethylamino)-1,6,10,11,12a-pentahydroxy-6-methyl-3,12-dioxo-N-(pyrrolidin-1-yl-methyl)-4,4a,5,5a-tetrahydrotetracene-2-carboxamide (Figure 6a) is a member of the first generation of tetracyclines characterized by high solubility in water. It was designed to be intravenously or intramuscularly injected. Tetracycline was combined with pyrrolidine, formaldehyde, and *tert*-butyl alcohol to obtain this derivative. Although it has been recommended when oral dosage forms are inappropriate, it is currently only used in exceptional cases [29].

*Eravacycline.* Eravacycline, (4*S*,4a*S*,5a*R*,12a*R*)-4-(dimethylamino)-7-fluoro-1,10,11,12a-tetrahydroxy-3,12-dioxo-9-[(2-pyrrolidin-1-ylacetyl)amino]-4a,5,5a,6-tetrahydro-4*H*-tetracene-2-carboxamide, is a synthetic fluorocycline created using a complete synthesis. Specific alterations were added to the naphtacen nucleus’ D ring. The D ring of eravacycline (the analog of tigecycline) has two essential modifications: the insertion of a fluorine atom in the C7 position, an electron-withdrawing substituent, and a pyrrolidin-acetamido group in the C9 position (Figure 6b). Eravacycline is very efficient against Gram-positive and Gram-negative bacteria, including resistant strains to tetracyclines. It was approved in 2018 to treat adults with complex intra-abdominal infections [37,75,76,77].

Eravacycline demonstrated an 8 to 16 times higher potency against *Klebsiella pneumoniae* and a 4 to 8 times higher potency against *Escherichia coli* when compared to tertiary alkylamine, dimethyl, azetidine, and piperidine counterparts. Eravacycline is also 4 to 64 times more effective than piperidine and azetidine homologs against tested bacterial isolates with known tetracycline-resistant genes *Enterococcus faecalis* [tet(M)], *Streptococcus pneumoniae* [tet(M)], *Escherichia coli* [tet(A)], and *Klebsiella pneumoniae* [tet(A)]), except (*Staphylococcus aureus* [tet(M) and tet(K)]. Compared to unsubstituted pyrrolidine analogs, adding polar substituents, fluorine atoms, or pyrrolidine bicycles did not result in any enhancements and had no adverse effects on the effectiveness against pneumococcal bacteria. The fluoro and pyrrolidine substitutions at C7 and C9 positions, respectively, had a beneficial impact on the antibacterial range and efficacy [78,79,80].

#### 4.1.6. Other Antibacterials

A new antibacterial compound is very close to receiving approval to treat infections. Therefore, we thought it appropriate for it to be mentioned here.

**Figure 7:**
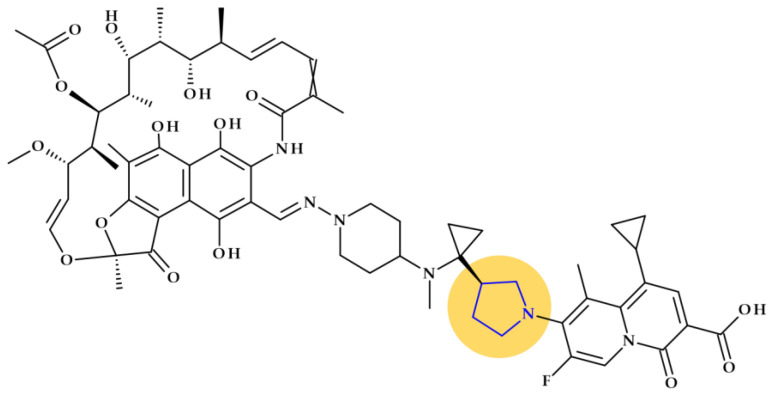
The molecular structure of Rifaquizinone (TNP-20292) with highlighted pyrrolidine heterocycle [82,83].

*Rifaquizinone* (TNP-2092). A rifamycin-quinolone hybrid compound with dual action (TNP-2092, formerly CBR-2092) was identified as a drug candidate currently in clinical development. The hybrid is 3-[(*E*)-[[4-[[1-[(3*R*)-1-(3-carboxy-1-cyclopropyl-7-fluoro-9-methyl-4-oxo-4*H*-quinolizin-8-yl)-3-pyrrolidinyl]cyclopropyl]methylamino]-1-piperidinyl]imino]methyl]rifamycin (Figure 7) [81,82,83]. Pyrrolidine compounds were used as starting materials for synthesizing the quinolizinone core (the lead ABT-714 compound) [81]. ABT-719 was previously synthesized at Abbott Laboratories [84]. TenNor Therapeutics (Suzhou, China) conducts clinical testing on TNP-2092, which completed Phase 2 for Acute Bacterial Skin and Skin Structure Infection (ABSSSI) (https://www.clinicaltrials.gov/show/NCT03964493, accessed on 24 October 2023). Both oral and intravenous routes are used to deliver the hybrid candidate [85,86,87].

### 4.2. Imidazole

Imidazole is a five-membered heterocyclic moiety with two double bonds, three carbon, and two nitrogen atoms, also known as 1, 3-diazole (Table 2). It has two nitrogen atoms, one of which has a hydrogen atom and the other is referred to as pyrrole-type nitrogen. The compound has in position 1 a pyrrolic-type nitrogen atom with acidic properties and in position 3 a pyridinic-type nitrogen atom with basic properties. Imidazole has acidic and basic properties due to its amphoteric nature [88]. There are two equivalent tautomeric forms of imidazole, and one of them allows the hydrogen atom to be located on one of the two nitrogen atoms [89].

The six extra electrons in this cycle are dispersed among the five other atoms but are mainly concentrated on the nitrogen atoms. This heterocycle has an excess of electrons. Positions 4 and 5 experience electrophilic replacements as a result of the electron excess, while position 2, where the electron density is lower, is susceptible to nucleophilic substitutions [22,24,25]. Despite electron density being concentrated on nitrogen atoms and classified as π-excessive heterocycle, the ring system’s electrons are delocalized. Easy protonation in strong acid, a sign of a strong base, is caused by a lone pair of electrons on N3. Also, a strong acid is indicated by the production of imidazolide in a strong base. As a result, imidazole has an amphoteric character [24]. The imidazole ring unsubstituted at the N1 position can be considered a weak acid. Considering these aspects, imidazole is amphoteric; it can function both as a base (iminic N3 atom) and as an acid (it can donate the proton from the secondary amino group (position N1)). Due to the abovementioned properties, imidazole can function as a hydrogen bond donor and acceptor, with the pyridinic N3 atom functioning as an electron pair donor and the N1 atom functioning as an acceptor. Thus, numerous mechanisms of action of some enzymes and drugs can be explained [22,89,90].

The imidazole can interact with numerous organic molecules via hydrogen bonds, van der Waals forces, ion-dipole, coordination, cation–π, π–π stacking, or hydrophobic effects [89]. Imidazole may create hydrogen bonds with its two nitrogen atoms, which increases its water solubility. The imidazole core’s strong polarity and capacity to complex various metal ions are two additional significant characteristics [22,25]. It serves as the fundamental building block of many natural products, including DNA-based structures, histidine, purines, and histamine [88].

Imidazole is a significant five-membered heterocycle in drug design. According to reports in the literature, the 1,3-diazole derivatives present a variety of biological activities [88,89]. Notably, a large number of imidazole-based compounds have been extensively used as clinical drugs, including anticancer, antifungal, antiparasitic, antihistaminic, anti-neuropathic, and antihypertensive medications, due to their high therapeutic potency and significant potential for future development [29,89].

#### 4.2.1. Macrolides (Ketolides Subclass)

**Figure 8:**
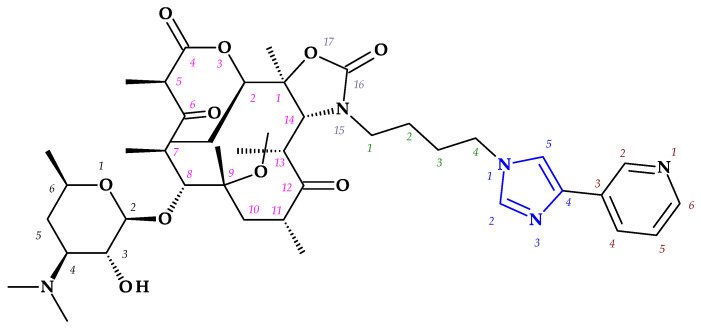
Telithromycin, chemical structure and atoms numbering by the IUPAC name.

*Telithromycin.* The macrolide class of antibiotics comprises the novel chemical class of ketolides, which includes the semisynthetic erythromycin derivative telithromycin (the first ketolide) [91]. Telithromycin, (1*S*,2*R*,5*R*,7*R*,8*R*,9*R*,11*R*,13*R*,14*R*)-8-[(2*S*,3*R*,4*S*,6*R*)-4-(dimethylamino)-3-hydroxy-6-methyloxan-2-yl]oxy-2-ethyl-9-methoxy-1,5,7,9,11,13-hexamethyl-15-[4-(4-pyridin-3-ylimidazol-1-yl)butyl]-3,17-dioxa-15-azabicyclo [12.3.0]heptadecane-4,6,12,16-tetrone, chemically differs from the macrolide group of antibacterials by the lack of α-*L*-cladinose at position 3 of the erythronolide A ring, resulting in a 3-keto function. This new ketolide lacks *L*-cladinose at position 3 of the erythronolide A ring and has a 3-keto action (differences from the macrolides group) [92]. Telithromycin has an additional pyridyl-imidazole-butyl side chain versus erythromycin. In telithromycin, the carbamate is linked to an alkyl-aryl extension, which gives the drug more potency than macrolides (Figure 8). As a result, telithromycin proved to be efficient against erythromycin-susceptible and resistant organisms, pneumococcus, as well against respiratory bacteria (*Haemophilus influenzae* and *Moraxella catarrhalis*) [91,93,94].

The mechanism of action of telithromycin is based on stopping bacterial growth by preventing their ability to synthesize proteins. Older macrolides only firmly bind to one domain of the 50S ribosomal subunit’s 23S RNA and weakly to the second domain, while telithromycin simultaneously strongly binds to both domains. Unfortunately, the presence of the pyridine-imidazole group of the telithromycin side chain was associated with uncommon but severe side effects (e.g., hepatotoxic effects) [94,95]. Consequently, to increase patient safety, the FDA (2007) announced a change to the telithromycin (Ketek^®^, Munich, Germany) use, the only indications being the treatment of CABP. In the European Union (EU), telithromycin was withdrawn from therapy [94,95,96].

#### 4.2.2. Nitroimidazoles

After azomycin (2-nitroimidazole) was isolated from streptomycetes (1953) and its anti-trichomonas action was demonstrated (1956), multiple chemical synthesis methods and biological experiments for other nitroimidazole compounds were carried out. Thus, 1-(β-hydroxyethyl)-2-methyl-5-nitroimidazole compound, also known as metronidazole, was discovered. Metronidazole proved activity in vitro and in vivo against the anaerobic protozoa *Trichomonas vaginalis* and *Entamoeba histolytica*. Later tests found that metronidazole was particularly effective in treating various anaerobic infections, including Gram-positive and Gram-negative bacteria and the protozoa *Giardia lamblia* [97].

**Figure 9:**
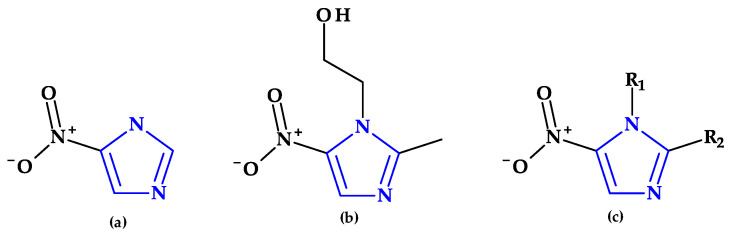
Chemical structures of (**a**) azomycin, (**b**) metronidazole, and (**c**) general chemical structure of 5-nitroimidazoles used in therapy.

Metronidazole, ornidazole, secnidazole, nimorazole, and tinidazole are examples of imidazole derivative drugs. These 5-nitroimidazole compounds (Figure 9) are frequently used in clinical settings to treat protozoa and anaerobic bacteria infections [29,89]. According to a theory, the fatal impact of pathogens is caused when a reactive intermediate created during the microbial reduction of the 5-nitro group of imidazole derivatives covalently attaches to the microorganism’s DNA. The nitroxide, nitroso, hydroxylamine, and amine are examples of potential reactive intermediates [29].

### 4.3. 2-Imidazolidinone

Found in antibacterial drugs, such as some beta-lactamase inhibitors, 2-Imidazolidinone is another five-membered heterocycle (Table 2).

#### Beta-Lactamase Inhibitors

**Figure 10:**
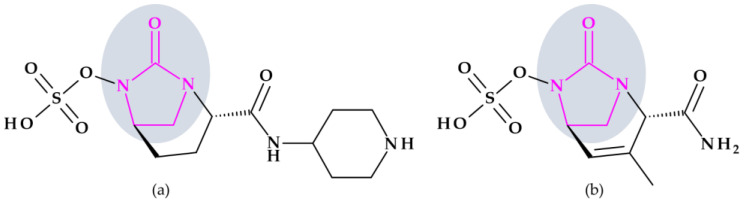
Chemical structure of beta-lactamase inhibitors with the highlighted 2-imidazolidinone heterocycle: (**a**) Relebactam and (**b**) Durlobactam.

*Relebactam.* Relebactam, [(2*S*,5*R*)-7-oxo-2-(piperidin-4-ylcarbamoyl)-1,6-diazabicyclo [3.2.1]octan-6-yl] hydrogen sulfate, is a beta-lactamase inhibitor that does not contain a beta-lactam ring. It is structurally similar to avibactam, with the only difference being the presence of a piperidine ring attached to the carbonyl group at the 2-position (Figure 10a) [98]. This new beta-lactamase inhibitor has been demonstrated to covalently bind and with high affinity to the active site of Ambler classes A and C serine beta-lactamases and specific class D enzymes [38]. Relebactam is a compound belonging to the diazabicyclooctane class of beta-lactamase inhibitors, similar to avibactam. The central diazabicyclooctane core comprises piperidine and a 2-imidazolidinone heterocycle, with two carbon and one nitrogen atom in common. The high reactivity of the diazabicyclooctane pharmacophore, which is influenced by the electron-withdrawing properties of an aminoxy-sulfate group and the strained nature of the bridged bicyclic urea core structure, contributes to its ability to greatly enhance the potency of beta-lactamase inhibition [38].

Recarbrio^®^ is a combination of imipenem (a carbapenem antibiotic), cilastatin (a renal dehydropeptidase inhibitor), and relebactam (a beta-lactamase inhibitor) approved by the FDA in 2019. This combination is prescribed to treat certain infections caused by susceptible Gram-negative bacteria, such as complicated UTIs (including pyelonephritis) and complicated intra-abdominal infections (cIAI), mainly when other treatment options are limited or unavailable. Moreover, it was also approved (in 2020) for the treatment of hospital-acquired bacterial pneumonia and ventilator-associated bacterial pneumonia (HABP/VABP) [99].

*Durlobactam.* Durlobactam, [(2*S*,5*R*)-2-carbamoyl-3-methyl-7-oxo-1,6-diazabicyclo [3.2.1]oct-3-en-6-yl] hydrogen sulfate, belongs to the diazabicyclooctane class of beta-lactamase inhibitors and exhibits wide-ranging effectiveness against Ambler class A, C, and D serine beta-lactamases. In contrast to the relebactam, the central diazabicyclooctane core comprises 1,2,3,6-tetrahydropyridine and a 2-imidazolidinone heterocycle, with two carbon and one nitrogen atom in common (Figure 10b) [100]. Unlike other beta-lactamase inhibitors, durlobactam’s molecular structure is characterized by endocyclic double bonds and methyl substituent. Durlobactam, a polar compound, can enter Gram-negative cells through outer membrane porins [101].

On 23 May 2023, the FDA approved a new drug called Xacduro^®^ for hospital-acquired bacterial pneumonia (HABP) and ventilator-associated bacterial pneumonia (VABP) cases. Specific strains of *Acinetobacter baumannii*-calcoaceticus complex produce these infections. Xacduro^®^ is composed of two components: sulbactam and durlobactam. Sulbactam, a beta-lactam antibiotic and a beta-lactamase inhibitor, is responsible for activity against *Acinetobacter baumannii* bacteria. At the same time, durlobactam protects sulbactam from being hydrolyzed by enzymes that the same bacteria may produce. It is administered through intravenous infusion [100].

### 4.4. 1,2,3-Triazole

**Figure 11:**
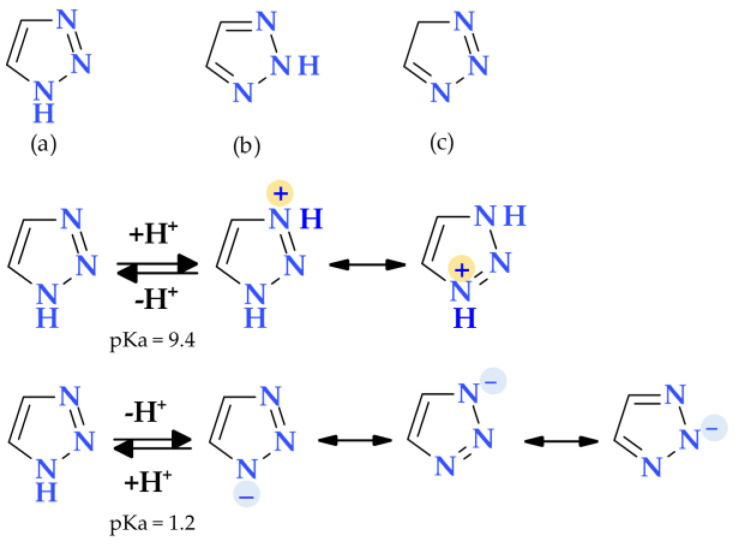
Tautomeric forms: (**a**) 1*H*-1,2,3-Triazole, (**b**) 2*H*-1,2,3-Triazole, (**c**) 4*H*-1,2,3-Triazole, and amphoteric behavior of 1,2,3-triazole [1,24].

The 1,2,3-triazole heterocycle is a planar, cyclic structure containing two carbon atoms and three nitrogen atoms (two are pyridinic and one is pyrrole type) (Table 2). There are three tautomeric forms of triazole—l*H*- and 2*H*- and 4-forms, the 2*H* form being the most stable. The 4*H*-1,2,3-triazole is a nonaromatic form. The 1*H*- and 2*H*-1,2,3-triazoles are in equilibrium in the gas phase and the solution. Electrophilic replacements at the level of the three nitrogen or carbon atoms are preferred in this heterocycle. Although 1,2,3-triazole is a weak base, it also has the strength of phenol when acting as a weak acid (Figure 11) [1,24].

The 1,2,3-triazole is aromatic because all of its atoms are sp2 hybridized, and its available six electrons are delocalized around the ring. The ionization energy of 1,2,3-triazole (10.06 eV) is greater than imidazole (8.78 eV) and pyrazole (9.15 eV). Although reductive cleavages are possible, the 1,2,3-triazole ring is exceedingly stable and can resist reductive, oxidative, and acidic/basic hydrolysis processes. Also, due to their aromatic nature, triazoles resist enzymatic degradation and participate in hydrogen bond formations, and dipole-dipole and π-stacking interactions. Due to the two nitrogen atoms in the ring that are of the pyridine type, quaternization is more complex and demands aggressive conditions [24,102,103]. Many standard and non-traditional methods have been used to synthesize 1,2,3-triazole derivatives [104]. Triazole can enhance a compound’s solubility and pharmacokinetic and pharmacodynamic features by creating hydrogen bonds, also known as dipole-dipole interactions. The result is the design of molecules with improved biological activity and selectivity [105,106].

#### 4.4.1. Beta-Lactamase Inhibitors

**Figure 12:**
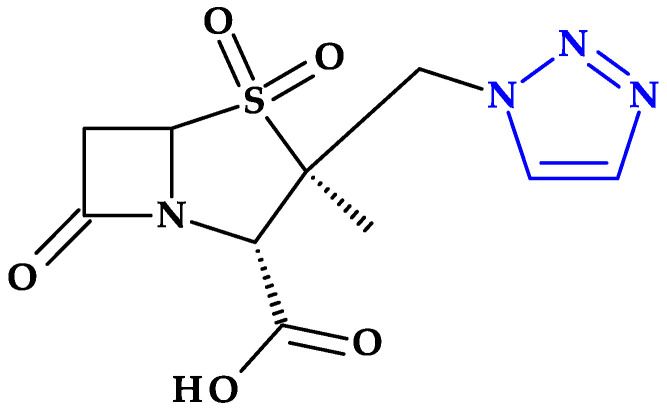
The chemical structure of tazobactam.

*Tazobactam.* Clavulanic acid was the first reported beta-lactamase inhibitor isolated in 1977 from *Streptomyces clavuligerus*. This was followed by the discovery of sulbactam and tazobactam in the 1980s. Sulbactam and tazobactam are penicillanic acid sulfones, whereas clavulanic acid is a clavam. Tazobactam, (2*S*,3*S*,5*R*)-3-methyl-4,4,7-trioxo-3-(triazol-1-ylmethyl)-4λ^6^-thia-1-azabicyclo [3.2.0]heptane-2-carboxylic acid, is considered the analog of sulbactam [107]. The heterocycle 1,2,3,-triazole is found in the structure of tazobactam (a beta-lactamase inhibitor) in the C3 position as 3-(triazol-1-ylmethyl) (Figure 12). 

In addition, tazobactam has a broader spectrum of activity than clavulanic acid (an oxapenam beta-lactamase inhibitor with (2-hydroxy ethylidene) moiety at C3). It is more effective than sulbactam (a beta-lactamase inhibitor with dimethyl substituents at C3) and other usual beta-lactamase inhibitors. Thus, its antibacterial activity is minimal [29,107].

### 4.5. Tetrazole

**Figure 13:**
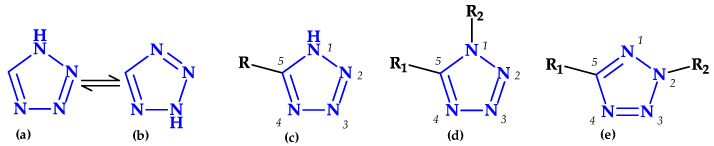
Tetrazole forms: (**a**) 1*H*-tautomer, (**b**) 2*H*-tautomer, (**c**) 5-monosubstituted, (**d**) 1,5-disubstituted, and (**e**) 2,5-disubstituted [24,109].

Tetrazole is a type of five-membered nitrogen heterocycle in which four nitrogen atoms are present next to each other and are connected by a single carbon bond and two double bonds (Table 2) [24]. Tetrazole presents two tautomeric forms, 1*H*-tetrazole and 2*H*-tetrazole, and is an aromatic aza pyrrolic system with six π delocalized electrons, and three pyridinic and one pyrrolic nitrogen atoms [1,24,108]. While the 2*H*-form is more stable in the gas phase, the 1*H*-form is more stable and appears more frequently in solution (Figure 13) [24].

A substantial electron-withdrawing inductive action is more significant than the mesomeric effect in the tetrazole heterocycle. The planar tetrazole anion has a strong aromatic property. As a result, it can contribute to the development of ion-dipole, ion-ion interactions with various electron-deficient compounds [1,109]. The unsubstituted tetrazole rings can establish intermolecular hydrogen bonds because the nitrogen atoms of the pyridinic type -N= act as proton acceptors. Some molecules’ structures allow the hydrogen atom, which comes from the pyrrolic type of nitrogen, to create intermolecular hydrogen bonds with specific electro-negative atoms. Despite having a weak basic nature, tetrazole can establish hydrogen bonds comparable to those formed by purine and pyrimidine bases [109].

Tetrazole is an acidic substance with a pKa of 4.89, similar to acetic acid’s pKa of 4.76 [24]. Tetrazolate anion has a planar structure and high aromaticity. This form can actively interact with partner molecules’ electron-deficient centers through ion-ion and ion-dipole interactions. Tetrazole rings that are NH-unsubstituted and 1,5-disubstituted are structural analogs of the carboxy and cisamide moieties that are metabolically stable [109]. 

Tetrazole is often used as a proline substitute due to increased solubility compared to proline [1]. Thereby, it is widely used as an isosteric replacement for carboxyl groups in the structure of several drugs due to the similarity to carboxylic acids and metabolic stability [1,25,109,110,111]. Also, tetrazole can interact with amidines at two points, like carboxylic acid. However, the tetrazole-amidine combination is less stable than the comparable carboxylate-amidine salt [111]. Tetrazole, as a bioisoster of natural amino acids and carboxylic acids, can enhance the pharmacokinetic profile of drugs with therapeutic efficacy by lowering their polarity and raising their lipophilicity for improved membrane permeability [109,111,112]. Tetrazole’s lack of involvement in phase II reactions during the metabolic process can be associated with the improvement of the pharmacokinetic profile of the new compound; it is advantageous for using it as a substitute for the carboxyl group to extend the compounds’ half-lives [113].

There are some tetrazole-based synthetic drugs from the classes of analeptics, antihypertensives, antifungals, antidiabetics, anti-inflammatories, anti-ulcers, metalloprotease inhibitors, growth hormone stimulators, opioid agonists, and chloride channel blockers used in clinical settings [24,29,114]. Also, a series of antibiotics containing the tetrazole heterocycle in their molecular structure are used in therapy [29].

#### 4.5.1. Beta-Lactam Antibiotics

**Cephalosporins**

**Figure 14:**
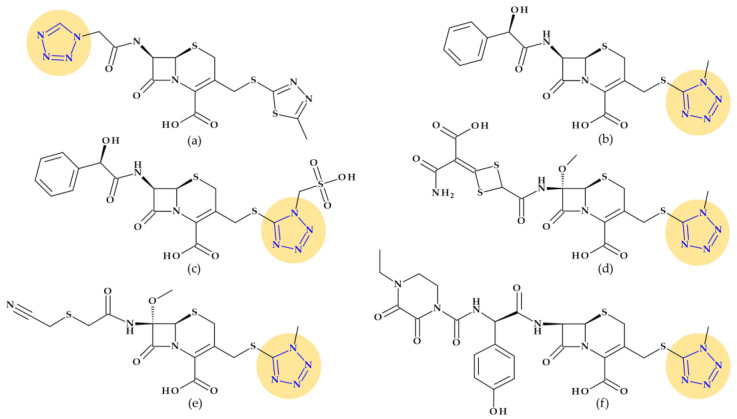
Cephalosporins whose chemical structure includes a tetrazole heterocycle: (**a**) Cefazolin (1st generation), (**b**) Cefamandole (2nd generation), (**c**) Cefonicid (2nd generation), (**d**) Cefotetan (3rd generation), (**e**) Cefmetazole (3rd generation), and (**f**) Cefoperazone (3rd generation) [29,31,117].

Cephalosporins with this moiety at the C3 position (such as cefamandole, cefmetazole, cefoperazone, cefotetan, and moxalactam) have been linked to a higher prevalence of hypoprothrombinemia in comparison to cephalosporins without the N-methyl-5-thiotetrazole group, by subsequently inhibiting glutamic acid’s gamma-carboxylation [29,115]. Another cephalosporin, cefazolin, presents a tetrazolyl acetylamino fragment at the C7 position (Figure 14), also associated with a higher prevalence of hypoprothrombinemia [29,115].

Additionally, this group has been linked to the alcohol intolerance produced by several injectable cephalosporins, including cefamandole, cefotetan, cefmetazole, and cefoperazone. As a result, patients who have consumed alcohol before, during, or after the beginning of therapy may experience disulfiram-like reactions that are attributed to acetaldehyde and brought on by the inhibition of the aldehyde dehydrogenase-catalyzed oxidation of ethanol by N-methyl-5-thiotetrazole-containing cephalosporins [29,31]. Cephalosporin-induced coagulation deficiencies or bleeding can be produced by several mechanisms such as: (a) the induction of vitamin K-responsive hypoprothrombinemia, (b) the production of an acquired platelet defect, and (c) thrombocytopenia secondary to bone marrow suppression, and others [116].

*Cefazolin* (1st generation). Cefazolin, (6*R*,7*R*)-3-[(5-methyl-1,3,4-thiadiazol-2-yl)sulfanylmethyl]-8-oxo-7-[[2-(tetrazol-1-yl)acetyl]amino]-5-thia-1-azabicyclo [4.2.0]oct-2-ene-2-carboxylic acid, is a semisynthetic cephalosporin approved in 1973 as a water-soluble sodium salt for parenteral administration. In addition to the 1,3,4-thiadiazole heterocycle at the C3 substituent, cefazolin contains an unusual tetrazolyl acetyl acylating moiety in the C7 position (Figure 14a) associated with a higher prevalence of hypoprothrombinemia [29,31,115]. Like first-generation cephalosporins, which demonstrated good action against Gram-positive bacteria and limited activity against Gram-negative bacteria, cephazolin’s activity spectrum and therapeutic uses are the same [117,118]. Cefazolin was often selected among the first-generation cephalosporins because its prolonged half-live (2.2 h) allows a more advantageous administration schedule [118].

*Cefamandole* (2nd generation). Cefamandole, (6*R*,7*R*)-7-[[(2*R*)-2-hydroxy-2-phenylacetyl]amino]-3-[(1-methyltetrazol-5-yl)sulfanylmethyl]-8-oxo-5-thia-1-azabicyclo [4.2.0]oct-2-ene-2-carboxylic acid, is a second-generation cephalosporin used as a nafate ester. Cefamandole nafate spontaneously hydrolyses to cefamandole at neutral to alkaline pH. Esterification of the α-hydroxyl group of the *D*-mandeloyl function overcomes the instability of cefamandole in solid-state dosage forms. It gives adequate concentrations of the parent antibiotic in vivo. The characteristic groups of cefamandole are a *D*-mandelic acid fragment and a (1-methyltetrazol-5-yl) sulfanyl methyl at the C3 position (Figure 14b). Also, cefamandole has a tetrazole-thiomethyl side chain associated with side effects. When combined with alcohol, cefamandole may (rarely) lengthen the prothrombin time and produces a disulfiram-like reaction [29,119]. Compared to the first-generation cephalosporins, cefamandole exhibits higher activity against Gram-negative bacteria [118]. 

*Cefonicid* (2nd generation). Cefonicid, (6*R*,7*R*)-7-[[(2*R*)-2-hydroxy-2-phenylacetyl]amino]-8-oxo-3-[[1-(sulfomethyl)tetrazol-5-yl]sulfanylmethyl]-5-thia-1-azabicyclo [4.2.0]oct-2-ene-2-carboxylic acid, is a second-generation cephalosporin with an almost similar structure to cefamandole. Compared to cefamandole, cefonicid has a methane sulfonic acid group linked to the N-1 position of the tetrazole ring (Figure 14c). Cefonicid and cefamandole have nearly equal antibacterial activity and limited lactamase stability. Thus, cefonicid has superior pharmacokinetic characteristics versus cefamandole. In comparison to other second-generation cephalosporins, cefonicid differentiates due to its relatively extended serum half-life of around 4.5 h [29,120,121]. The pharmacological action and indications for use are analogous to those of cefamandole [117].

*Cefotetan* (3nd generation). Third-generation cephalosporin cefotetan, (6*R*,7*S*)-7-[[4-(2-amino-1-carboxy-2-oxoethylidene)-1,3-dithietane-2-carbonyl]amino]-7-methoxy-3-[(1-methyltetrazol-5-yl)sulfanylmethyl]-8-oxo-5-thia-1-azabicyclo [4.2.0]oct-2-ene-2-carboxylic acid, is unaffected by beta-lactamases due to a methoxy moiety at the C7 position of the cephalosporanic system (a cephamycin antibiotic). Also, the 1-methyltetrazol-5-yl group in cefotetan (Figure 14d) has been linked to hypoprothrombinemia and alcohol intolerance [29,31,119]. Third-generation cephalosporins are usually less effective than first-generation representatives against Gram-positive cocci. However, they are substantially more effective against Enterobacteriaceae despite beta-lactamase-producing strains, causing a significant increase in resistance [118].

*Cefmetazole* (3rd generation). Another third-generation cephalosporin for parenteral use is cefmetazole, (6*R*,7*S*)-7-[[2-(cyanomethylsulfanyl)acetyl]amino]-7-methoxy-3-[(1-methyltetrazol-5-yl)sulfanylmethyl]-8-oxo-5-thia-1-azabicyclo [4.2.0]oct-2-ene-2-carboxylic acid. This cephalosporin belongs to the cephamycin family. Like other cephamycins, the C7-methoxy group offers resistance to various beta-lactamases. However, the methyl tetrazole moiety (Figure 14e) has been linked to increased bleeding in some high-risk patients, similar to other cephalosporins [29,122].

*Cefoperazone* (3rd generation). Cefoperazone, (6*R*,7*R*)-7-[[(2*R*)-2-[(4-ethyl-2,3-dioxopiperazine-1-carbonyl)amino]-2-(4-hydroxyphenyl)acetyl]amino]-3-[(1-methyltetrazol-5-yl)sulfanylmethyl]-8-oxo-5-thia-1-azabicyclo [4.2.0]oct-2-ene-2-carboxylic acid, exhibits chemical and biological properties with piperacillin (a broad-spectrum beta-lactam antibiotic of the ureidopenicillin class) [29]. Additionally, cefoperazone contains a side chain of methyl thiotetrazole (Figure 14f) and can lead to bleeding, especially when taken in quantities larger than 4 g daily [115,116]. Cefoperazone is efficient against *Pseudomonas aeruginosa* but less efficient against Gram-positive cocci than other third-generation representatives [118].

**Oxacephalosporins**

**Figure 15:**
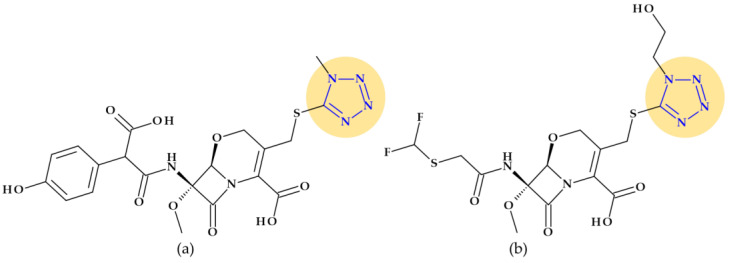
Oxacephalosporins the chemical structure of which includes a tetrazole heterocycle: (**a**) Latamoxef (1st generation), and (**b**) Flomoxef (2nd generation) [29,123,124].

*Latamoxef*/*Moxalactam* (1st generation). Latamoxef, (6*R*,7*R*)-7-[[2-carboxy-2-(4-hydroxyphenyl)acetyl]amino]-7-methoxy-3-[(1-methyltetrazol-5-yl)sulfanylmethyl]-8-oxo-5-oxa-1-azabicyclo [4.2.0]oct-2-ene-2-carboxylic acid, is an oxacephalosporin from the first generation, a cephamycin antibiotic (with a C7-methoxy moiety). The oxygen atom in the 7-aminocephalosporanic acid nucleus of latamoxef replaced the sulfur atom in the nucleus of the genuine cephalosporins (Figure 15a) [123]. Latamoxef more frequently induces coagulopathy and bleeding than other cephalosporins [116]. Like cefamandole, it has an N-methylthiotetrazole side-chain and may cause hypoprothrombinaemia [115,123].

Thereby, latamoxef has been linked to severe bleeding episodes. Therefore, prophylaxis with vitamin K and monitoring of bleeding time has been indicated throughout therapy. Inhibition of platelet function and, less frequently, immune-mediated thrombocytopenia may also interfere with hemostasis and hypoprothrombinemia. Also, a disulfiram-like reaction to alcohol is possible, similar to cephalosporins that include the methylthiotetrazole heterocycle [123]. These potentially fatal side effects ultimately led to Latamoxef medication being discontinued [125]. Identical to the third-generation cephalosporin cefotaxime, latamoxef exhibits antibacterial activity, albeit it tends to be less effective against Gram-positive bacteria and more effective against *Bacteroides fragilis* [123].

*Flomoxef* (2nd generation). Flomoxef, ((6*R*,7*R*)-7-[[2-(difluoromethylsulfanyl)acetyl]amino]-3-[[1-(2-hydroxyethyl)tetrazol-5-yl]sulfanylmethyl]-7-methoxy-8-oxo-5-oxa-1-azabicyclo [4.2.0]oct-2-ene-2-carboxylic acid) [126], is also an oxacephalosporin from the second generation, similar to latamoxef [29,123]. This antibiotic includes an extra C7-β-difluoromethyl-thioacetamido side chain substitution (Figure 15b), resulting in significant activity levels against Gram-positive and Gram-negative bacteria (including anaerobes) and a lower toxicity profile. In addition, the N-methyl tetrazole thiol group linked to the C3 position of the oxacephem nucleus is replaced with a methyl thiadiazole-thiol group, which is presumed to be the cause of the disulfiram- and coumarin-like adverse effects associated with latamoxef therapy [124].

#### 4.5.2. Oxazolidinones

**Figure 16:**
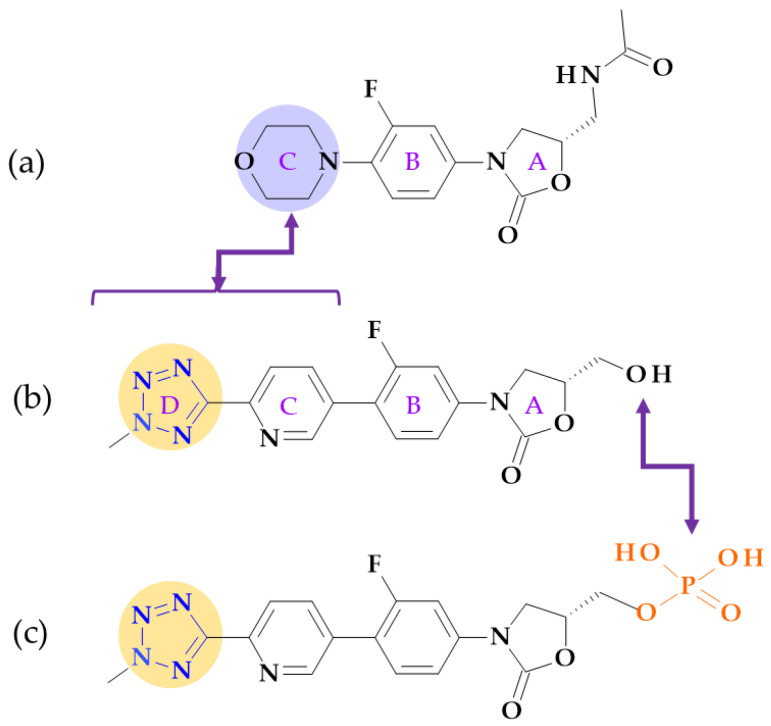
Structural differences between (**a**) linezolid, (**b**) tedizolid, and (**c**) tedizolid phosphate; the morpholine ring in linezolid was replaced with a pyridine and a methyl tetrazole ring in tedizolid [31,128,130].

*Tedizolid* (2nd generation). In 2014, the FDA approved tedizolid, (5*R*)-3-{3-fluoro-4-[6-(2-methyl-2*H*-1,2,3,4-tetrazol-5-yl)pyridin-3-yl]phenyl}-5-(hydroxymethyl)-1,3-oxazolidin-2-one [127], a second-generation oxazolidinone synthesized as the phosphate prodrug, for treating acute bacterial skin and skin structure infections (ABSSSIs) produced by susceptible Gram-positive organisms, such as MRSA. Compared to linezolid, tedizolid is 4- to 16-fold more effective against MRSA [110,128]. The optimizations of linezolid (the first approved oxazolidinone) by replacing the morpholine ring with a pyridine one and an additional methyl tetrazole ring were responsible for the enhanced potency of tedizolid (Figure 16) [128,129].

Due to these structural optimizations, tedizolid can engage in additional binding interactions with the upper region of the peptidyltransferase center of the 50S ribosomal subunit, inhibiting protein synthesis. Tedizolid’s pyridine and methyl tetrazole rings add two hydrogen bonds to the residues A2451 and U2584 of the sugar backbone. The antimicrobial potency of tedizolid has been linked to the formation of more target site interactions at the peptidyltransferase center [128,129].

It is well known that several prodrugs containing tetrazole heterocycle as a bioisoster of carboxylic acid have received enhanced oral bioavailability, lipophilicity, and bioavailability while lowering its adverse effects [110]. Given that tedizolid has a mean half-life of 12 h, which is twice as long as linezolid’s (5.4 h), it can be used once daily, which is more convenient. Tedizolid has an excellent pharmacokinetic profile, a half-life for once-daily treatment, and absent steady-state nonlinearities. The administration of tedizolid phosphate can be undertaken regardless of food [130,131,132]. In addition to more favorable pharmacokinetics and enhanced antimicrobial potency, tedizolid phosphate proves a lower incidence of side effects, including thrombocytopenia [130].

Although it is in development (two phase 2 clinical trials), we consider it appropriate to discuss the following compound that has the potential to be shortly approved.

**Figure 17:**
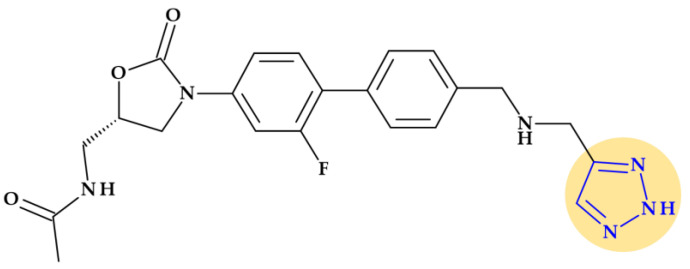
Chemical structure of radezolid with highlighted triazole heterocycle.

*Radezolid.* Melinta scientists developed the second-generation oxazolidinone antibacterial drug radezolid, N-[[(5S)-3-[3-fluoro-4-[4-[(2*H*-triazol-4-ylmethylamino)methyl]phenyl]phenyl]-2-oxo-1,3-oxazolidin-5-yl]methyl]acetamide, to increase ribosomal binding affinity and lower off-target activity [21,133]. In Figure 17, it is shown that the molecular building blocks of radezolid are the triazole ring, methylamino methyl link, biaryl ring system, oxazolidinone ring, and acetamide fragment. Radezolid is entirely synthesized and has one stereocenter in the oxazolidinone ring (position C5) [134]. Abscesses, bacterial skin illnesses, streptococcal infections, infectious skin disorders, and staphylococcal skin infections have all been studied in clinical trials with radezolid [21,133]. Two phase 2 clinical trials were completed to treat pneumonia and uncomplicated skin infections [135], [136]. This new oxazolidinone derivative possesses enhanced antibacterial potency against antibiotic-resistant Gram-positive bacteria compared with linezolid. Its action mechanism consists of forming π-π stacking interactions with the 50S subunit of the bacteria [137].

## 5. Five-Membered Heterocycles Containing Oxygen Atoms

### 5.1. Furan

The word furan, which indicates bran, is derived from the Latin *furfur* [24]. Furan is an aromatic five-membered heterocycle with a centrally positioned sp2 hybridized oxygen atom that is planar and pentagonal (Table 2). Furan’s ring atoms all lay in a plane, forming a pentagon with minor distortion. The bond length between C-3 and C-4 is more extended than between C-2 and C-3 and between C-4 and C-5. Therefore, the C-C bond length averages the single and double bond lengths, while the C-O bond is shorter by 0.05 Å. The C-C bond length is approximately the same (1.33 Å). The places next to the heteroatom were previously denoted as α and α’. Furyl is the name attributed to the monovalent residue [1,22,24].

One of the two pairs of non-participating electrons is in an sp2 hybridized orbital, while the other pair is in a π orbital. These two pairings are in separate orbitals. The ring’s bonds are comparable to those in the pyrrolic ring, and the heterocycle displays six delocalized electrons [1]. Furan reacts with electrophilic reagents similarly to benzene, frequently with substitution. However, depending on the reagent and reaction circumstances, it can also react via addition and/or ring-opening [22]. Furan is faster than benzene to participate in electrophilic substitution reactions because it is a heterocycle with excess electrons. Therefore, furan is more reactive than thiophene but less reactive than pyrrole in reactivity [1,22]. Thus, furan is less stable than thiophene due to its lower resonance energy [24].

Primarily, furan serves as a precursor in synthesizing and manufacturing numerous chemical components necessary for the synthesis of drugs [24]. Several drugs from different therapeutic classes contain the furan heterocycle in their chemical structure: 5-nitrofuran derivatives and cefuroxime (antibacterials), darunavir (HIV protease inhibitor), furosemide (diuretic), lapatinib (antineoplastic agent and tyrosine kinase inhibitor), prazosin (alpha-blocker used to treat hypertension), and ranitidine (histamine H2 antagonist) [24,29,31,138].

Drug discovery frequently uses structural alerts to detect compounds likely to produce harmful metabolites. Le Dang N. et al. (2017) showed that mathematical models of P450 metabolism can forecast the context-specific possibility that a structural alarm will be biologically activated in a particular molecule. Furan heterocycles were found in 17 approved or withdrawn drugs. Among these, thirteen were in the literature-derived Accelrys Metabolite Database, and three were bioactivated through epoxidation. In this study, three 5-nitrofuran derivatives (furazolidone, nitrofural, and nitrofurantoin) and a cephalosporin (cefuroxime) were targeted from the class of antibiotics. None of these compounds showed metabolism by epoxidation [139].

#### 5.1.1. Beta-Lactam Antibiotics

**Cephalosporins**

**Figure 18:**
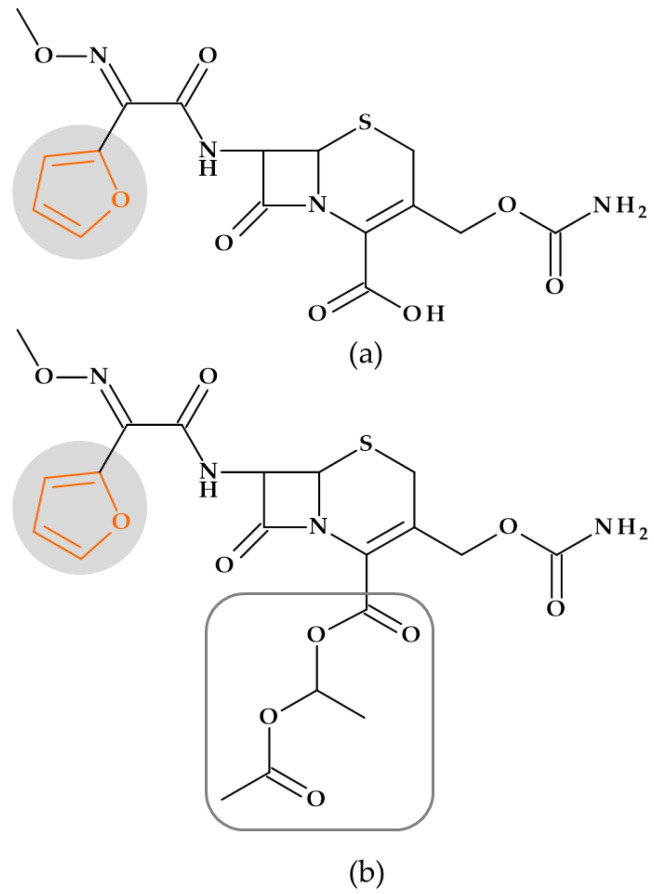
Cephalosporins: (**a**) Cefuroxime and (**b**) Cefuroxime axetyl (acetyoxyethyl ester of cefuroxime), whose chemical structure include a furan heterocycle.

*Cefuroxime*/*Cefuroxime axetil* (2nd generation). Cefuroxime, (6*R*,7*R*)-3-(carbamoyloxymethyl)-7-[[(2*Z*)-2-(furan-2-yl)-2-methoxyiminoacetyl]amino]-8-oxo-5-thia-1-azabicyclo [4.2.0]oct-2-ene-2-carboxylic acid, is a second-generation cephalosporin with a similar antibacterial activity spectrum to cefamandole [29]. The C-7 position of cefuroxime has a *Z*-oriented methoxy imino moiety into a (2*Z*)-2-(furan-2-yl)-2-methoxyiminoacetyl]amino fragment, which confers significant resistance from attack by many but not all beta-lactamases [31,140]. The 1-acetyoxyethyl ester of cefuroxime is known as cefuroxime axetil (Figure 18). This lipophilic, acid-stable oral prodrug derivative of cefuroxime is hydrolyzed to cefuroxime during intestinal and/or plasma enzyme absorption [29]. *Escherichia coli*, *Klebsiella pneumoniae*, *Neisseria gonorrhoea*, and *Haemophilus influenzae* are beta-lactamase-producing bacteria resistant to cefamandole and susceptible to cefuroxime. Thus, Serratia and Proteus species (indole-positive), *Pseudomonas aeruginosa*, and *Bacteroides fragilis* are Gram-negative pathogens resistant to cefuroxime [31].

#### 5.1.2. Nitrofurans

**Figure 19:**
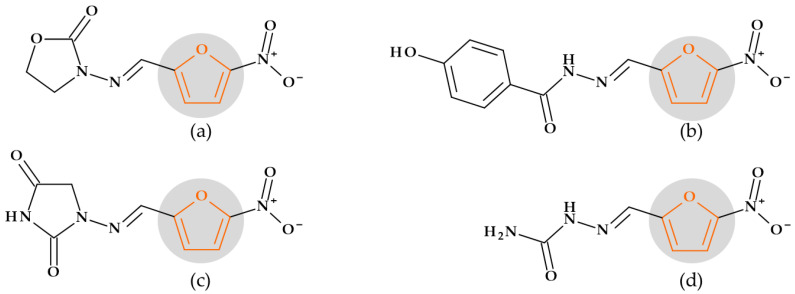
Nitrofurans, the chemical structure of which includes a furan heterocycle: (**a**) Furazolidone, (**b**) Nifuroxazide, (**c**) Nitrofurantoin, and (**d**) Nitrofurazone.

According to chemical structure–biological activity relationship studies, the 5-nitrofural element and the azomethine group are essential for nitrofurans’ antibacterial, antifungal, and antiprotozoal activity. In addition, the rest of the heterocyclic amine determines its activity spectrum and pharmacokinetic properties (Figure 19) [29]. Nitrofurans exhibit activity against Gram-positive and Gram-negative bacteria, particularly the Enterobacter, Citrobacter, Escherichia, and Klebsiella species. Additionally, it shows antiprotozoal and antimycotic effects against *Giardia lamblia* and *Trichomonas vaginalis*, respectively. Nitrofurans’ mode of action is based on the production of superoxide ions, nitroso derivatives, and highly reactive free radical species that result from the activity of certain reductases in bacterial cells. The bacterial ribosomal proteins will attach to the compound produced by reductases. As a result, bacterial nucleic acid structure and function, or bacterial metabolism, are affected [29,118].

#### 5.1.3. Oxazolidinones

Although the following compound is an investigational drug, this may be a “lead” compound for other oxazolidinones.

**Figure 20:**
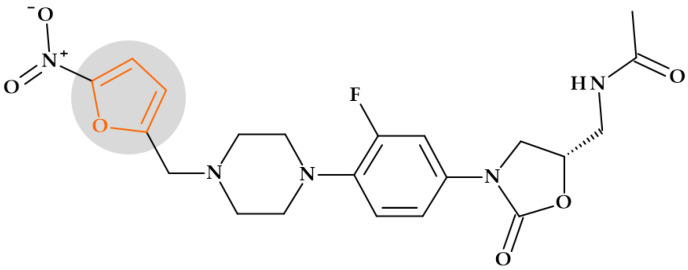
Ranbezolid, the chemical structure of which includes a furan heterocycle.

*Ranbezolid.* The ‘piperazinyl-phenyl-oxazolidinone’ core of eperezolid was used to synthesize new oxazolidinones that had a variety of substituted five-membered heterocycles connected to it. These optimizations resulted from several compounds with strong action against various resistant and sensitive Gram-positive pathogens [141]. Thus, a 2-substituted-5-nitro-furyl derivative was identified as ranbezolid (RBx 7644) with the IUPAC name N-[[(5S)-3-[3-fluoro-4-[4-[(5-nitrofuran-2-yl)methyl]piperazin-1-yl]phenyl]-2-oxo-1,3-oxazolidin-5-yl]methyl]acetamide (Figure 20) [141]. The compound in this group with the highest in vivo activity was ranbezolid, which also had in vitro activity comparable with or slightly better than linezolid. Additionally, ranbezolid is active against Gram-positive and Gram-negative anaerobes [141,142].

## 6. Five-Membered Heterocycles Containing Oxygen and Nitrogen Atoms

### 6.1. 1,3-Oxazolidine

Oxazolidine is a five-membered heterocycle with two heteroatoms, one represented by oxygen in position 1 and the other by nitrogen in position 3 (Table 2). There are two isomers of this heterocycle: 1,3-oxazolidine and 1,2-isoxazolidine. Tetrahydro-oxazole, or oxazole, is represented by the heterocycle in its fully reduced state [24]. 1,3-Oxazolidine-2-one derivatives were created and developed to cure infections produced by Gram-positive bacteria resistant to different antibiotics. First, the antibacterial properties of linezolid and other oxazolidinone compounds that prevented bacterial protein synthesis were discovered [143].

#### Oxazolidinones

In 1987, oxazolidinones (DuP 721 and DuP 105) were introduced as a new class of antibiotics [144]. Currently, they are used in therapy with two representatives: linezolid (1st generation) and tedizolid (2nd generation). The five-membered heterocycle oxazolidine, which has a 2-oxazolidinone carbonyl group attached (A cycle), is the pharmacophore for this class of drugs (Figure 16) [145]. The 1,3-oxazolidin-2-onic nucleus represents oxazolidinone’s basic structure, essential to antibacterial activity [146]. Thus, the C5 acyl aminomethyl group, the 5*S* configuration, and the N-aryl substituent were all necessary for antibacterial properties. Although it was not necessary, the meta-fluoro substitution of the phenyl ring typically increased antibacterial activity, and the para substitution might be modified to broaden the antibacterial spectrum [147].

Oxazolidinone antibiotics act by inhibiting the formation of the initiation complex, which inhibits the start of bacterial protein synthesis. The 30S and 50S ribosomal subunits, rRNA, and mRNA are part of the initiation complex. The 50S ribosomal subunit’s peptidyl-transferase enzyme center can interact with the 1,3-oxazolidin-2-onic ring. The peptidyl transferase enzyme is the catalytic center and plays a fundamental role in bacterial protein synthesis [145,146,148].

Oxazolidinones are a relatively recent class of antibiotics developed to mainly treat Gram-positive bacterial infections [149]. They are active on a broad spectrum of aerobic and anaerobic Gram-positive bacteria, including bacteria resistant to other antibiotics - methicillin- and vancomycin-resistant staphylococci, vancomycin-resistant enterococci, penicillin-resistant pneumococci, and *Mycobacterium tuberculosis* [118,146].

Linezolid is the “first-in-class” representative of oxazolidinones approved by the FDA in 1999 [145] for treating skin infections, nosocomial pneumonia, and drug-resistant tuberculosis [149]. Therefore, infections caused by *Enterococcus faecalis*, pneumonia caused by *Staphylococcus aureus* and *Streptococcus pneumoniae*, and complicated skin infections caused by methicillin-resistant *Staphylococcus aureus* and *Staphylococcus pyogenes* are all treated with linezolid [150]. Tedizolid phosphate is the inactive prodrug of tedizolid [147] approved by the FDA in 2014 for treating skin and skin structure infections [128,130,147]. Both oxazolidinones were previously discussed in Section 4.5.2. regarding structural features correlated with pharmacological and toxicological aspects. 

Notably, other oxazolidinones such as posizolid, sutezolid, radezolid, eperezolid, delpazolid, torezolid, and TBI-223 are in different phases of clinical trials [146].

### 6.2. Oxazoles and Isoxazoles

With five atoms, including the heteroatoms oxygen and nitrogen, oxazole is an unsaturated molecule belonging to the 1,3 azole class (Table 2) [1]. Because a -CH= group was substituted for an azomethine group, which has a nitrogen atom of the pyridinic type, it is regarded as a furan derivative; this is considered a heterocycle since it has an excess of electrons and an aromatic π sextet [1,24]. However, the existence of an electronegative oxygen atom limits the total delocalization of electrons. Since the oxazole’s nitrogen atom exhibits pyridine-type behavior and its oxygen atom exhibits furan-type behavior, it is regarded as a hybrid heteroaromatic system [24]. It has been noticed that the aromaticity order is comparable to that of the five-membered heterocycles with a single heteroatom (thiophene > pyrrole > furan). Among the 1,3-azoles, oxazole has the least aromatic content and has diene-like characteristics. Following the bond lengths in oxazole (with a noticeable difference in the C2-N and C4-N bonds), the oxazole ring is planar and has significant bond fixation [1].

Isoxazoles are an essential type of five-membered aromatic heterocycles that include three conventional sp2 carbon atoms and two electronegative heteroatoms, nitrogen and oxygen, in a 1,2 relationship (Table 2) [24]. Isoxazole is a monocyclic heteroarene (1-oxa-2-azacyclopentadiene). It is a heterocycle with excess electrons and an oxygen atom of the furan type in position 1 and a nitrogen atom of the pyridinic type in position 2. As a result, it will develop features unique to those two types of atoms [1].

Isoxazole is a π-excessive heterocycle with characteristics similar to pyridine and furans. The electrophilic substitution of isoxazole develops more frequently than pyridine due to the electron-attracting quality of the pyridine type of nitrogen and the electron-donating characteristic of oxygen. Isoxazoles can be modified to produce a variety of complex structures. As oxazole, it is an aromatic system, and the oxygen and nitrogen heteroatoms in the five-membered ring significantly impact the aromatic feature. Various reactions, including electrophilic substitution, nucleophilic substitution, oxidation, reduction, etc., are carried out in this heteroaromatic system [24].

Oxazole is regarded as a hybrid of both heterocyclic systems. It demonstrates characteristics of both (i) pyridine-type nitrogen, which includes protonation, N-alkylation, and the reactivity of the halogen atom at position 2, and (ii) furan-type oxygen, which includes diene-type characteristics as a result of bond localization [1]. Similar to imidazoles, oxazoles are weakly basic compounds that can be either partially reduced (2,5-dihydro oxazole or 4,5-dihydro oxazole) or totally reduced (oxazolidine) [24]. 

Because it is capable of producing weak non-covalent bonds, including hydrogen bonds, ion-dipole interactions, and van der Waals interactions, the oxazole nucleus has the potential to be biologically active. Oxazole is used to structure several compounds with pharmacological effects because it binds to receptors and interacts with various enzymes [24]. Also, the isosteres of oxazole are thiazole, imidazole, benzimidazole, triazole, and tetrazole. The pharmacokinetic profile of multiple drugs is enhanced, their range of activity is expanded, and their toxicity is decreased due to oxazole heterocycle in the molecular structure [151].

Oxazole has been identified as a critical molecular building block for developing new drugs. Numerous structurally complex natural compounds have this ring system. Oxazole-containing drugs have been obtained from marine and plant sources [24]. Oxazoles, in particular, can bind with a wide range of enzymes and biological receptors and exhibit a wide range of pharmacological activities, being included in antibacterial, antifungal, antiviral, antitubercular, anticancer, and anti-inflammatory medicines [24,151]. Isoxazoles have also been discovered to be essential components in many synthetic items used daily and as a pharmacophore present in many medications and bioactive natural products. Additionally, isoxazoles have shown they can interact with several enzymes and receptors in hydrogen bond donor/acceptor interactions. Isoxazoles are helpful building blocks for many different compounds, including many natural compounds, when synthetically created [24].

#### 6.2.1. Beta-Lactam Antibiotics

**Isoxazolyl penicillins (Antistaphylococcal penicillins)**

The beta-lactam cycle is hydrolyzed by bacteria into inactive molecules by hydrolytic enzymes known as beta-lactamases (penicillinases). Researchers made structural modifications at the level of the radical linked to the carbonyl bond to avoid this phenomenon. The carbonyl group is shielded and given steric hindrance by adding a sizeable heterocyclic residue (e.g., isoxazole) [29]. The bioisosteric substitution of the benzene ring (isoxazolyl) in benzylpenicillin has resulted in the biosynthesis of isoxazolyl penicillins. Chemical differences between isoxazolyl penicillins include fluorine or chlorine substituents on the benzene ring [31]. Including the isoxazole heterocycle in their molecular structure improves beta-lactamase resistance and resistance in the acidic stomach environment [29,118].

**Figure 21:**
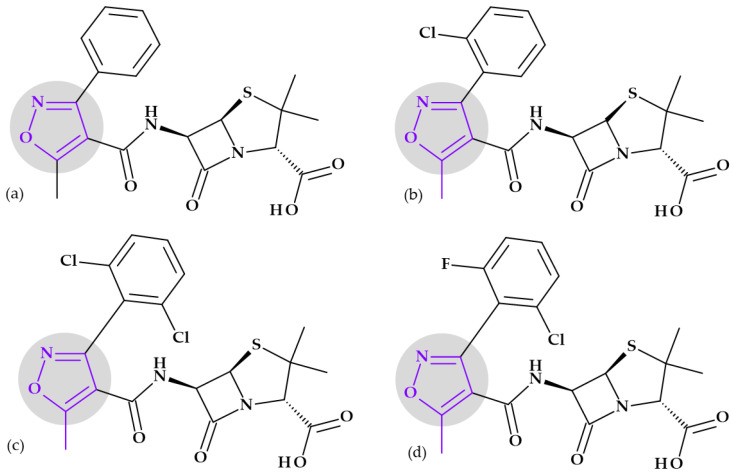
Isoxazolyl penicillins including 1,2-oxazole heterocycle in their molecular structures: (**a**) Oxacillin, (**b**) Cloxacillin, (**c**) Dicloxacillin, and (**d**) Flucloxacillin.

Oxacillin and its derivatives, cloxacillin, dicloxacillin, and flucloxacillin (Figure 21), are rarely used in therapy. Pulcini et al. (2012) named these antibiotics “forgotten antibiotics”. Unfortunately, economic motives are the primary cause for the discontinuation of the marketing of these antibiotics [13].

Isoxazolyl penicillins are typically less effective than benzylpenicillin against Gram-positive pathogens (staphylococci and streptococci) that do not develop a beta-lactamase. Still, they retain their effectiveness against those that do. Since isoxazolyl penicillins are more acid-stable than natural penicillins, they can be orally administered and have a higher potency. However, they are poor options for treating septicemia due to their high serum protein binding. The isoxazolyl group of penicillins are highly lipophilic compounds active against bacteria resistant to methicillin and methicillin-sensitive *Staphylococcus aureus* (MSSA). Due to their inability to pass through Gram-negative bacteria’s cell walls, these antibiotics have a limited spectrum of activity. Penicillins from the isoxazolyl group are mainly utilized to treat infections with *Staphylococcus aureus*, which produces beta-lactamase [29,31,118,152]. Although their spectrum of activity is comparable to benzylpenicillin, isoxazolyl penicillins should be restricted to treating infections induced by Staphylococci resistant to benzylpenicillin [153].

#### 6.2.2. Izoxazolidinones

**Figure 22:**
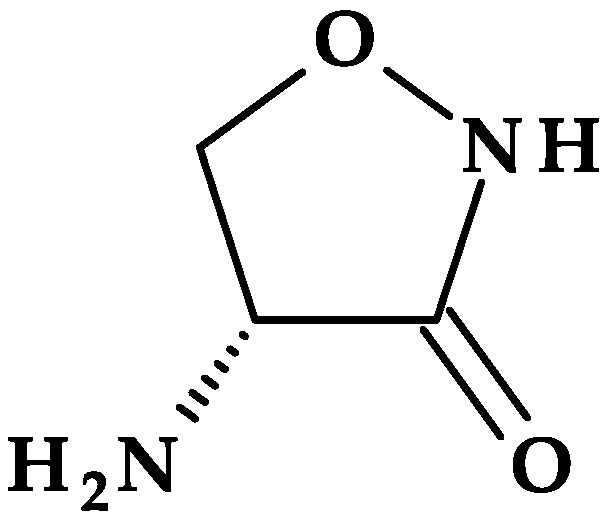
Chemical structure depiction of Cycloserine.

*Cycloserine.* Cycloserine, *D*-(+)-4-amino-3-isoxazolidinone, is an antibiotic from the isoxazolidinones class and second-line therapy for tuberculosis [31,154]. The antibiotic has been isolated from three different Streptomyces species’ fermenting beer (Figure 22). Later, it was synthesized, being a relatively simple structure. The stereochemistry of cycloserine and *D*-serine is similar. However, the *L*-form exhibits comparable antibiotic action [29].

As a mechanism of action, cycloserine inhibits the synthesis of the bacterial wall. The inhibition occurs in the early phase of peptidoglycan synthesis, a cell wall constituent [31,154]. The peptidoglycan of the mycobacterial cell wall contains a significant amount of *D*-alanine. *D*-alanine racemase is an enzyme that converts naturally available *L*-alanine to D-alanine in Mycobacteria [31]. The enzymes alanine racemase and *D*-alanyl-*D*-alanyl synthetase are involved in the production of the dipeptide *D*-alanyl-*D*-alanine, a precursor of the pentapeptide chain in the development of mycobacterial cell walls. Both enzymes are inhibited by cycloserine. It has been suggested that cycloserine has a better probability of binding to the enzyme because the isoxazole ring has a more rigid structure than *D*-alanine (a more flexible structure) [154]. So, the rigid analog *D*-cycloserine competitively inhibits *D*-alanine binding to both of these enzymes and, consequently, its inclusion into the peptidoglycan [31].

Despite antibiotic efficacy in vitro against various Gram-positive and Gram-negative pathogens, cycloserine is limited to treating tuberculosis due to its relatively low potency and frequent side effects due to the excellent penetration of cerebrospinal fluid [29,155,156]. A series of central nervous system (CNS) side effects are due to the binding of *D*-cycloserine to neuronal N-methylasparate (NMDA) receptors and affecting the synthesis and metabolism of γ-aminobutyric acid (GABA) [31,155].

Some antibacterials in development, such as posizolid and zoliflodacin, include in their molecular structure an isoxazole heterocycle. 

**Figure 23:**
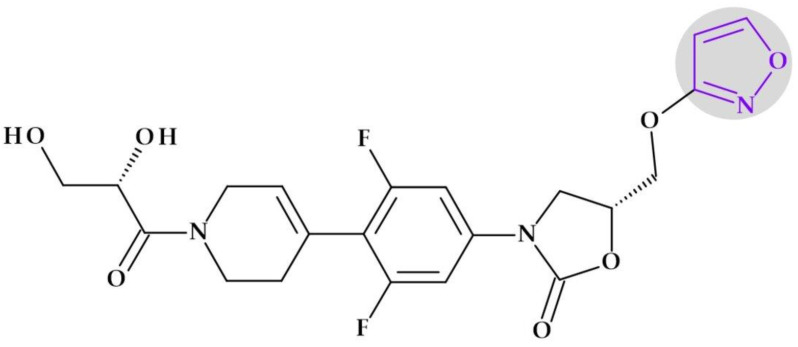
Chemical structure depiction of Posizolid.

*Posizolid.* Posizolid, (5*R*)-3-[4-[1-[(2S)-2,3-dihydroxypropanoyl]-3,6-dihydro-2*H*-pyridin-4-yl]-3,5-difluorophenyl]-5-(1,2-oxazol-3-yloxymethyl)-1,3-oxazolidin-2-one, includes an extra 1,2-oxazole heterocycle in its molecular structure in addition to the oxazolidinone nucleus (Figure 23); it is in phase I of clinical trials [149].

**Figure 24:**
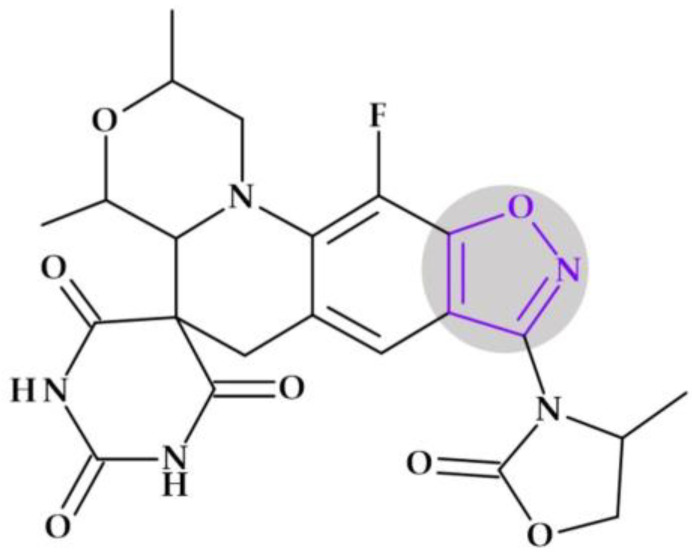
Chemical structure depiction of Zoliflodacin.

*Zoliflodacin.* The novel antibacterial class spiro-pyrimidinetrione includes zoliflodacin, 4′*R*,6′*S*,7′*S*)-17′-fluoro-4′,6′-dimethyl-13′-[(4*S*)-4-methyl-2-oxo-1,3-oxazolidin-3-yl]spiro [1,3-diazinane-5,8′-5,15-dioxa-2,14-diazatetracyclo [8.7.0.02,7.012,16]heptadeca-1(17),10,12(16),13-tetraene]-2,4,6-trione, as its first representative. Zoliflodacin contains the spirocyclic pyrimidinetrione pharmacophore. Izoxazole is present in the molecular structure of this promising novel antibiotic currently undergoing a global phase 3 randomized controlled trial to treat gonorrhea (Figure 24) [157,158].

#### 6.2.3. Sulfonamides

Antibacterial sulfonamides are bacteriostatic agents that work as antimetabolites of para-aminobenzoic acid (PABA), which competitively inhibit dihydropteroate synthetase, an enzyme involved in the synthesis of bacterial folic acid [31]. Microorganisms considered sensitive must produce folic acid; bacteria that can utilize preformed folate are unaffected. Sulfonamides are bacteriostatic when used alone; nevertheless, complete infection eradication requires the host’s cellular and humoral defensive mechanisms [118]. Some sulfonamides contain 1,2-oxazole heterocycle in their chemical structure, such as sulfisoxazole and sulphamethoxazole.

**Figure 25:**
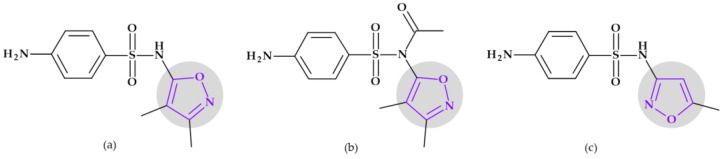
Sulfonamides including 1,2-oxazole heterocycle in their molecular structures: (**a**) Sulfisoxazole, (**b**) Sulfisoxazole acetyl, and (**c**) Sulfamethoxazole.

*Sulfisoxazole* (*Sulfafurazole*). Sulfisoxazole, 4-amino-N-(3,4-dimethyl-1,2-oxazol-5-yl)benzenesulfonamide, or sulfafurazole contains a 1,2-oxazole heterocycle in its chemical structure (Figure 25a). In the studies of the structure-activity relationship (SAR), replacing one of the amino function hydrogens with an electron-withdrawing heteroaromatic ring (1,2-isoxazole) enhanced the remaining hydrogen’s acidity and potency. Sulfisoxazole has a pKa of approximately 5.0. It is a short-acting sulfonamide, the half-life of sulfisoxazole being about 5 to 8 h. It enters the fetal circulation through the placenta and is released into the breast milk. Sulfisoxazole treats infections caused by sulfonamide-sensitive bacteria since it has the same effects and applications as other sulfonamides. Gram-negative urine infections have responded well to this sulphonamide therapy [29,31,123].

*Sulfisoxazole diolamine*. The 2,2′-iminodiethanol (diolamine) salt of sulfisoxazole is prepared (1:1 ratio) to make the sulfonamide more soluble in the physiological pH. When it cannot be maintained by oral treatment, the diolamine salt is used in solution for systemic delivery by slow intravenous, intramuscular, or subcutaneous injection. Additionally, it is utilized for applying drops or ointments to the eye to treat localized infections that are susceptible to sulfisoxazole [29].

*Sulfisoxazole acetyl.* Like its parent chemical, sulfisoxazole, sulfisoxazole acetyl (Figure 25b) exhibits the same properties and applications. Since the acetyl derivative has no taste, it can be orally administered, especially in liquid solutions. The acetyl molecule functions as a prodrug for sulfisoxazole, splits in the intestinal system, and is absorbed as sulfisoxazole. It is essential to distinguish between acetyl sulfafurazole and the N4-acetyl derivative of sulfafurazole produced through conjugation in the body [29,123].

*Sulfamethoxazole.* In terms of chemical structure and antibacterial activity, sulfamethoxazole, 4-amino-N-(5-methyl-1,2-oxazol-3-yl)benzenesulfonamide, is a sulfonamide medication that is related to sulfisoxazole. The difference is given by the methyl substituent on the oxazole ring and the 3-position of the heterocycle linked to the sulfonamide amino (N1) group (Figure 25c). The plasma half-life of sulfamethoxazole is 6 to 12 h, longer than that of sulfisoxazole (about 6 h) [29,123].

The sulfamethoxazole and trimethoprim combination (cotrimoxazole) represented a significant step forward in the design of antimicrobial drugs that were both clinically efficacious and synergistic. Trimethoprim inhibits the bacterial enzyme dihydrofolate reductase from converting dihydrofolate to tetrahydrofolate [118,159]. The sulfamethoxazole and trimethoprim combination can be bactericidal because it inhibits two processes in the bacterial biosynthesis of vital proteins and nucleic acids. Gram-positive and Gram-negative bacteria, Nocardia, *Chlamydia trachomatis,* and certain protozoa are all inhibited by sulfonamides. Also, some enteric bacteria are inhibited, including *Escherichia coli*, Klebsiella, Salmonella, Shigella, and Enterobacter species. Unfortunately, many previously susceptible species’ strains have developed resistance. Among the FDA-approved indications are acute infective exacerbation of chronic bronchitis, otitis media (only in children), prevention and treatment of travelers’ diarrhea, UTIs, Shigellosis, prophylaxis, and therapy of *Pneumocystis pneumonia* and Toxoplasmosis [29,159].

## 7. Five-Membered Heterocycles Containing One Sulfur Atom

### 7.1. Tiophene

Thiophene is a five-membered unsaturated aromatic heterocycle often referred to as furan’s sulfur analog (Table 2) [1]. The sulfur atom’s presence has a specific impact on the aromatic nature of the compound as well as its characteristics and reactions. In the π electron system, sulfur’s “electron pairs” are strongly delocalized and behave as highly reactive, similar to a benzene derivative [160].

Thiophene exhibits an electron cloud, making it similarly reactive to benzene. Due to the weakly electronegative sulfur atom, it is the least reactive to the action of electrophilic agents when compared to furan and pyrrole. However, compared to benzene, its electrophilic substitutions occur significantly more quickly [1,161]. The bioisosterism link between thiophene and benzene is explained by the discovery of thiophene as an impurity in benzene and the remarkable similarity between the two chemicals’ physicochemical features. Therefore, thiophene can be effectively used to replace the benzene nucleus in the structure of molecules of medicinal interest as a bioequivalent [160].

Chemical structures known as structural alerts or toxicophores can be bioactivated to produce reactive metabolites. Le Dang N. et al. (2017) indicated that the thiophene structural alert is unclear. Currently, it is known that thiophenes can be bioactivated by epoxidation. Different oxidative metabolic processes can convert thiophenes into electrophilic, unstable intermediates such as S-oxides, epoxides, and sulfenic acids. Toxicity can result from the oxidative metabolism of thiophenes, which produces reactive, electrophilic intermediates [139].

Thiophene is found in various natural compounds, such as biotin (vitamin H) [1]. The biological activity of thiophene and its derivatives include antiviral, antifungal, antibacterial, antileishmanial, antimicrotubule, anti-inflammatory, antioxidant, anticancer, and anti-HIV effects. Because of these effects, thiophene is a critical structural component in several therapeutically relevant drugs [161,162].

#### Beta-Lactam Antibiotics

**Carboxypenicillins**

The presence of an extra carboxyl group at the acylamino group (at the C6 position) of these semisynthetic penicillins led to the generic name of carboxypenicillins. Compared to other penicillins, the carboxyl moiety enhances the molecule’s ability to penetrate through Gram-negative bacilli’s cell wall barriers. Carboxypenicillins are typically used to treat infections produced by Gram-negative, ampicillin-resistant bacilli [29]. Two carboxypenicillins containing thiophene in the molecular structure are ticarcillin and their analog, temocillin.

**Figure 26:**
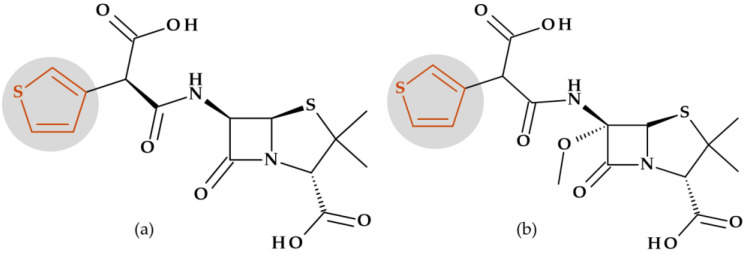
Carboxypenicillins including thiophene heterocycle in their molecular structures: (**a**) Ticarcillin and (**b**) Temocillin.

*Ticarcillin.* Ticarcillin, (2*S*,5*R*,6*R*)-6-[[(2*R*)-2-carboxy-2-thiophen-3-ylacetyl]amino]-3,3-dimethyl-7-oxo-4-thia-1-azabicyclo [3.2.0]heptane-2-carboxylic acid, is an isostere of carbenicillin in which a thiophene heterocycle substitutes the phenyl. It is also known as α-carboxy-3-thienylpenicillin (Figure 26a) and is formulated as a disodium salt. Because this semisynthetic penicillin derivative is unstable in acid, parenteral administration is required [29]. Ticarcillin is a fourth-generation extended-spectrum penicillin previously used to treat mild to severe infections caused by sensitive Gram-positive and Gram-negative organisms. Ticarcillin’s broad spectrum made it a suitable antibiotic against *Pseudomonas aeruginosa*. Additionally, ticarcillin exhibits efficacy against a few Enterobacter and Proteus species. Ticarcillin is active against most bacteria susceptible to natural penicillins but is frequently less effective [163].

This semisynthetic penicillin is less effective than piperacillin (a ureidopenicillin) but more effective than carbenicillin against *Pseudomonas aeruginosa*. A combination of ticarcillin and clavulanate has been used to treat intra-abdominal UTIs and has activity against Gram-negative aerobic and anaerobic pathogens. Currently, ticarcillin and its combination with clavulanate are no longer manufactured [118]. In 2004, ticarcillin was withdrawn from the United States (US) market [163].

*Temocillin.* In the 1980s, the semisynthetic 6-methoxy derivative of ticarcillin, known as temocillin, (2*S*,5*R*,6*S*)-6-[(2-carboxy-2-thiophen-3-ylacetyl)amino]-6-methoxy-3,3-dimethyl-7-oxo-4-thia-1-azabicyclo [3.2.0]heptane-2-carboxylic acid (Figure 26b), was commercialized in Belgium and the United Kingdom [164]. Temocillin was immediately discontinued because of what was considered to be significant limitations, including a lack of action against anaerobes, *Pseudomonas aeruginosa*, and Gram-positive pathogens [12]. Although temocillin is an old antibiotic, given its unique properties, it may be an efficient substitute for carbapenems when treating infections caused by Enterobacteriaceae that produce extended-spectrum beta-lactamase (ESBL) and uncomplicated UTIs caused by carbapenemase-producing *Klebsiella pneumoniae* (KPC) producers [164].

**Cephalosporins**

**Figure 27:**
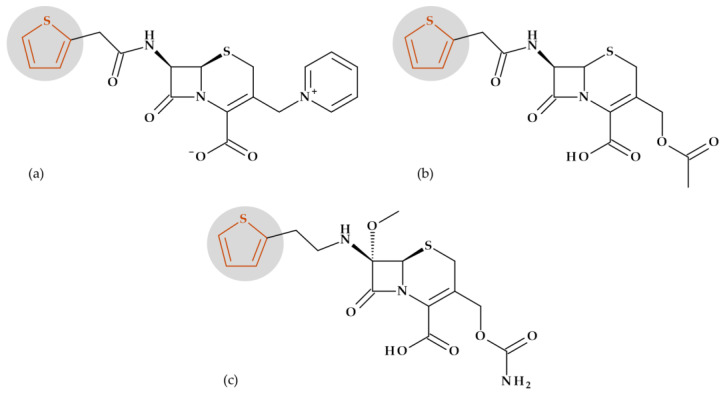
Cephalosporins including thiophene heterocycle in their molecular structures: (**a**) Cephaloridine, (**b**) Cephalotine, and (**c**) Cefoxitin.

*Cephaloridine (Cefaloridine)* (1st generation). Cephaloridine, (6R,7R)-8-oxo-3-(pyridin-1-ium-1-ylmethyl)-7-[(2-thiophen-2-ylacetyl)amino]-5-thia-1-azabicyclo [4.2.0]oct-2-ene-2-carboxylate (Figure 27a) was the first successful semisynthetic cephalosporin [165]. Cephaloridine has properties comparable to cephalotin, briefly discussed below [123]. Thus, cefaloridine was the first cephalosporin with noticeable dose-related nephrotoxicity. Cephaloridine builds up in the proximal renal tubular cell, most likely through active anionic transport [166]. It is not available for therapeutic purposes due to the availability of newer cephalosporins and concerns about potential side effects and bacterial resistance [123].

*Cephalothin* (*Cefalotin*) (1st generation). The first-generation cephalosporins have moderate effectiveness against Gram-negative bacteria but considerable efficacy against Gram-positive bacteria [118]. Structurally, cephalothin is 7-(thiophene-2-acetamido) cephalosporanic acid, respectively (6*R*,7*R*)-3-[(acetyloxy)methyl]-8-oxo-7-[2-(thiophen-2-yl)acetamido]-5-thia-1-azabicyclo [4.2.0]oct-2-ene-2-carboxylic acid (Figure 27b) [167]. In the presence of sodium bicarbonate, cephalothin was produced through the direct reaction of the 2-thienylacetic acid chloride with 7-aminocephalosporanic acid [117]. The spectrum of activity of cephalothin is more comparable to ampicillin than benzylpenicillin. Cephalothin, in contrast to ampicillin, is resistant to the penicillinase produced by *Staphylococcus aureus*, offering an option for the use of penicillins that are penicillinase-resistant for the treatment of infections produced by such strains [29]. Cephalothin has been used to treat various blood, bone or joints, respiratory tract, skin, and UTI infections and to avoid infection during surgery [117,168]. In therapy, it has currently been replaced by new generations of cephalosporins.

*Cefoxitin* (2nd generation). Among beta-lactamase–resistant 7-α-methoxy cephalosporins (cephamycins) is cefoxitin, (6*R*,7*S*)-3-(carbamoyloxymethyl)-7-methoxy-8-oxo-7-[(2-thiophen-2-ylacetyl)amino]-5-thia-1-azabicyclo [4.2.0]oct-2-ene-2-carboxylic acid [29], a compound that contains a thiophene heterocycle in the side chain from the C6 position (Figure 27c). One of the first cephamycins available was cefoxitin (as sodium salt) [123]. The ability of cefoxitin (and cephamycins in general) to kill resistant bacterial strains is due to the 7-α-methoxyl substituent’s protection against hydrolysis by beta-lactamases. Even though cefoxitin is less effective than cephalothin against Gram-positive bacteria and cefamandole against the majority of Enterobacteriaceae, it is nevertheless effective against some strains of Gram-negative bacteria that are resistant to these cephalosporins. *Neisseria gonorrhoeae* and penicillin-resistant *Staphylococcus aureus* are also resistant to it. A disadvantage of cefoxitin is its short half-live (about one hour), and therefore, cefoxitin needs to be parenterally given three to four times daily [29,123].

## 8. Five-Membered Heterocycles Containing Sulfur and Nitrogen Atoms

### 8.1. 1,3-Thiazolidine

1,3-Thiazolidine is a saturated five-membered heterocycle with sulfur and nitrogen in positions 1 and 3, respectively (Table 2); it is considered an analog of oxazolidine [161]. When thiazolidines are present in an acidic or basic aqueous solution, they hydrolyze to form aldehyde and amino thiol. The production of an iminium thiolate zwitterion intermediate has led researchers to conclude that the reaction involves breaking the C-S bond. Thiazolidines can be hydrolyzed to aldehydes under neutral conditions with metal ions such as Hg(II) or Cu(II). The existence of this moiety in penicillin derivatives was the main motive for the mechanistic research of thiazolidine ring opening [169].

#### Beta-Lactam Antibiotics: Natural and Semisynthetic Penicillins

**Figure 28:**
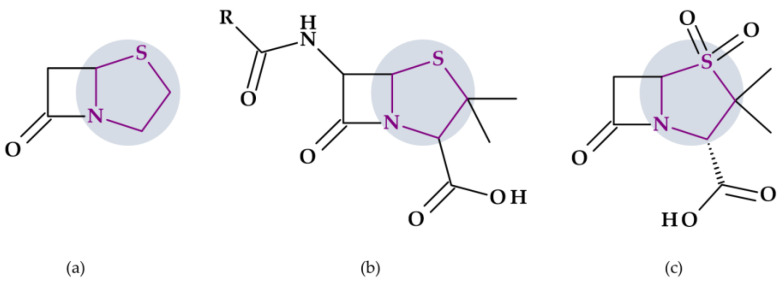
The thiazolidine heterocycle (purple) depiction in (**a**) Penam bicycle, (**b**) Penicillins molecular structure, and (**c**) Sulbactam.

The penicillin antibiotics contain a fused ring system, a substituted five-membered thiazolidine ring fused to the beta-lactam ring (cyclic amide with four atoms) [29,31], essential for antibacterial activity. This heterocycle proved to be the main component of the pharmacophore [31]. Penicillin’s unsubstituted bicyclic ring structure is named “penam” because it makes it simpler to comprehend (Figure 28a). The penicillins are referred to as 4-thia-1-azabicyclo (3.2.0) heptanes (Figure 28b) [153].

Sulbactam, (2*S*,5R)-3,3-dimethyl-4,4,7-trioxo-4λ^6^-thia-1-azabicyclo [3.2.0]heptane-2-carboxylic acid, is penicillanic acid sulfone. The 1,3-thiazolidine 1,1-dioxide is a fragment of the central core of sulbactam, which replaced thiazolidine in the “penam” heterocycle of penicillins (Figure 28c) [104]. 

Sulbactam is a potent inhibitor of beta-lactamases produced by *Staphylococcus aureus* and Gram-negative bacilli. While it has limited antibacterial activity, it enhances the effectiveness of ampicillin and carbenicillin against beta-lactamase-producing *Staphylococcus aureus* and certain species in the Enterobacteriaceae family. However, it does not synergize with carbenicillin or ticarcillin on *Pseudomonas aeruginosa* strains resistant to these drugs [29].

Xacduro^®^ (sulbactam for injection and durlobactam for injection), a recent drug approved by the FDA in 2023, was previously discussed in Section 4.4.1. Sulbactam is responsible for activity against *Acinetobacter baumannii*, whereas durlobactam protects sulbactam from being degraded by beta-lactamases that may be produced by *Acinetobacter baumannii* [100].

### 8.2. 1,3-Thiazole

With one sulfur atom and one nitrogen atom of the pyridine type at position 3 of the cyclic ring system, thiazole is a five-membered, unsaturated, planar, and excessive heteroaromatic (Table 2) [1,22]. Thiazole is believed to be generated from thiophene by substituting a pyridine-type nitrogen (azomethine) at position 3 for the -CH= group. The structure of thiazole is quite comparable to the average of the structures of thiophene and 1,3,4-thiadiazole, although it is predicted to be structurally related to thiophene and pyridine. Therefore, it is anticipated that the chemical reactions of thiazole will be comparable to those of pyridine and thiophene [1] due to the presence of thiophene-type sulfur at position 1 and pyridine-type nitrogen at position 3 of the thiazole ring. Electrophilic reactions have three possible sites: sulfur, nitrogen, and the C5 position. Though, the electronically weak site C2 is vulnerable to nucleophilic assault [22].

Thiazole is considered a planar, aromatic ring due to the delocalization of a pair of nonparticipating electrons from the sulfur atom (position 1). It has an excess of π electrons concentrated on the heteroatoms. Given this, electrophilic attacks will occur at the 1, 3, 4, and 5 positions, and nucleophilic attacks will appear at the C2 position [22,170,171]. According to molecular orbital calculations, the thiazole molecule is aromatic with some dienic characteristics. Thiazole’s ring current corresponds to its aromatic nature [1].

Considering acid-base properties, thiazole (pKa of 2.5) is a weak base compared to pyridine (pKa of 5.2) but is more basic than oxazole (pKa of 0.8) [1]. The compound is miscible with water. It can form stable salts with strong acids by protonating the imine nitrogen (position 3) [25,161]. Thiazole is a substance that is highly stable and does not autoxidize. By protonating the imine nitrogen, thiazole forms stable crystalline salts with strong acids known as thiazolium salts [25].

There are many natural substances having thiazole rings exhibiting diverse biological properties. For example, vitamin B1 (thiamine) has a thiazole ring connected to 2-methylpyrimidine-4-amine [22]. Additionally, many thiazole synthetic derivatives have pharmacological activity. Numerous thiazole-containing drugs are successfully utilized in therapy [172]. Other compounds are in various stages of development [172,173,174,175]. Among antibacterials, several beta-lactam antibiotics and sulphonamides include thiazole in their molecular structure [1,172].

#### 8.2.1. Beta-Lactam Antibiotics

**Cephalosporins**

Cephalosporins’ aminoacyl moiety appears to become more stable against some beta-lactamases’ action when polar substituents are added [29]. Various heterocycles associated with better pharmacokinetics and antibacterial activity can take place in the side chain of cephalosporins; amino-thiazoles are among these (Table 5) [153,172]. Unusual, cefditoren, a third-generation cephalosporin, contains an extra thiazole heterocycle at the C3 position as a 4-methyl-1,3-thiazol moiety. The antibacterial activity of cefditoren is similar to cefixime. In addition, cefditoren is efficient against *Staphylococcus aureus* [123].

**Monobactams**

Unlike penicillins and cephalosporins, which fuse the four-membered antibacterial beta-lactam structure to another heterocyclic ring, monobactams contain unfused beta-lactam heterocycle. It represents the most basic beta-lactam with antibacterial properties [165]. Including an aminothiazole oxime side chain as the 3-acyl substituent, typical to several third-generation cephalosporins, they have resulted in the most notable increase in Gram-negative antibacterial activity. This fragment is responsible for the drugs’ remarkable activity against Gram-negative bacteria through increased affinity for their PBPs. Aztreonam was the first monobactam generated through side-chain modifications [177]. Further, several antibacterial drugs with the (*S*)-3-[2-(2-aminothiazol-4-yl)-2-(oxyimino) acetamido]-2-oxoazetidine-1-sulfonate structure were developed. To further widen the antibacterial activity and improve the stability of beta-lactamase hydrolysis, several modifications have been added at the 3- or 4-position of the beta-lactam ring or the oxime side chain [177].

**Figure 29:**
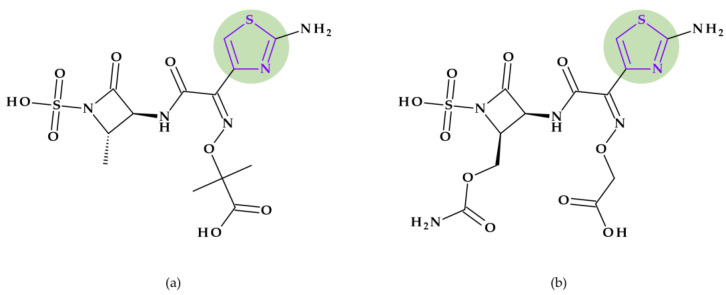
The thiazole heterocycle (purple) depiction in (**a**) Aztreonam and (**b**) Carumonam molecular structure.

*Aztreonam.* The FDA and European regulatory agencies approved the monobactam antibiotic aztreonam in 1986 [178]. Aztreonam, 2-[(*Z*)-[1-(2-amino-1,3-thiazol-4-yl)-2-[[(2S,3S)-2-methyl-4-oxo-1-sulfoazetidin-3-yl]amino]-2-oxoethylidene]amino]oxy-2-methylpropanoic acid, has been created using a total synthesis [29]. Antibacterial spectrum and resistance to beta-lactamases are determined by the 3-side chain amino thiazolyl oxime moiety and the 4-methyl group (Figure 29a) [29]. Aztreonam only presents a strong affinity for PBP 3 in Gram-negative bacteria but has no activity against Gram-positive bacteria and anaerobes. Beta-lactamase resistance is similar to ceftazidime’s, which possesses the same isobutyric acid oximinoacyl moiety [29]. Thus, the beta-lactamases with a broad spectrum hydrolyze aztreonam [165]. In the past, aztreonam was mainly used to treat infections of the lower respiratory tract, the urinary tract, and intra-abdominal infections, as well as septicemia, endometritis, pelvic cellulitis, and infections of the skin and skin structures caused by aerobic Gram-negative bacteria [178]. Although it is considered an old antibiotic, aztreonam has become a particular area of interest when combined with beta-lactamase inhibitors and other antimicrobials [179].

*Carumonam.* Carumonam, 2-[(*Z*)-[1-(2-amino-1,3-thiazol-4-yl)-2-[[(2S,3S)-2-(carbamoyloxymethyl)-4-oxo-1-sulfoazetidin-3-yl]amino]-2-oxoethylidene]amino]oxyacetic acid, is an N-sulfonated monobactam (Figure 29b) that is relatively stable against beta-lactamases. When a carbamoyloxymethyl group was added in a 3,4-cis configuration at the 4-position, the antibacterial activity against Gram-negative bacteria, including strains that produce beta-lactamases, was greatly improved. It has a similar broad antibacterial spectrum to aztreonam against common bacteria like Enterobacteriaceae, *Pseudomonas aeruginosa*, and *Haemophilus influenzae* [177,180]. When those pathogen agents produce respiratory or urinary tract infections, carumonam is a therapy option [180].

Some monobactams containing 1,3-thiazole, such as pirazmonam and tigemonam, are under development.

**Figure 30:**
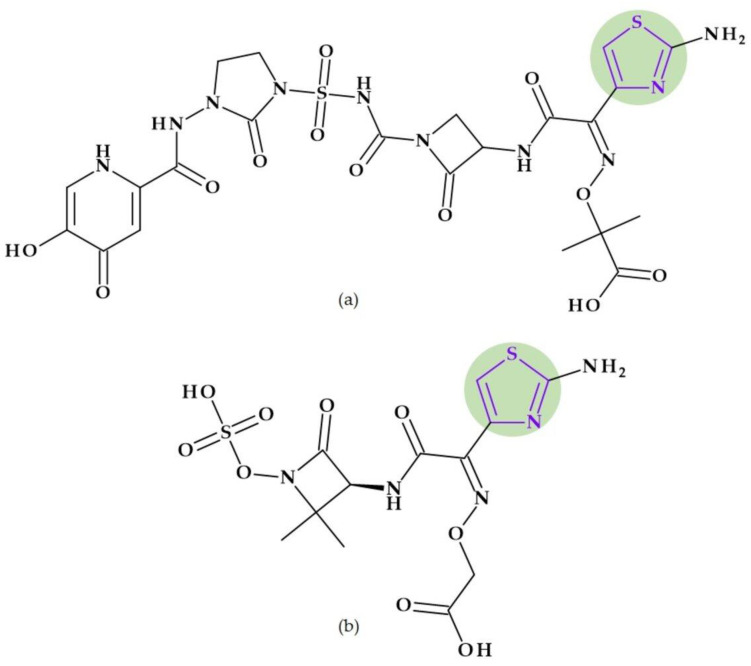
The thiazole heterocycle (purple) depiction in (**a**) Pirazmonam and (**b**) Tigemonam molecular structure.

*Pirazmonam.* The first monocyclic β-lactam-siderophore conjugate prepared from the monobactams was pirazmonam, 2-[(*Z*)-[1-(2-amino-1,3-thiazol-4-yl)-2-[[1-[[3-[(5-hydroxy-4-oxo-1H-pyridine-2-carbonyl)amino]-2-oxoimidazolidin-1-yl]sulfonylcarbamoyl]-2-oxoazetidin-3-yl]amino]-2-oxoethylidene]amino]oxy-2-methylpropanoic acid (Figure 30a). The new compound had a 3-hydroxy-4-pyridone iron-chelating fragment included in the N1-activating group. Thus, it retained many beneficial properties characteristic of aztreonam but had significantly improved activity against *Pseudomonas aeruginosa* [177].

*Tigemonam.* Tigemonam, 2-[(*Z*)-[1-(2-amino-1,3-thiazol-4-yl)-2-[[(3S)-2,2-dimethyl-4-oxo-1-sulfooxyazetidin-3-yl]amino]-2-oxoethylidene]amino]oxyacetic acid (Figure 30b), is an oral monobactam; its oral absorption is excellent in comparison to the poor oral bioavailability of aztreonam. Moreover, it exhibits a strong resistance against beta-lactamases. Tigemonam demonstrates a similar antibacterial effectiveness as aztreonam. However, it is not highly effective against Gram-positive or anaerobic bacteria and has no activity against *Pseudomonas aeruginosa* [29].

#### 8.2.2. Sulphonamides

**Figure 31:**
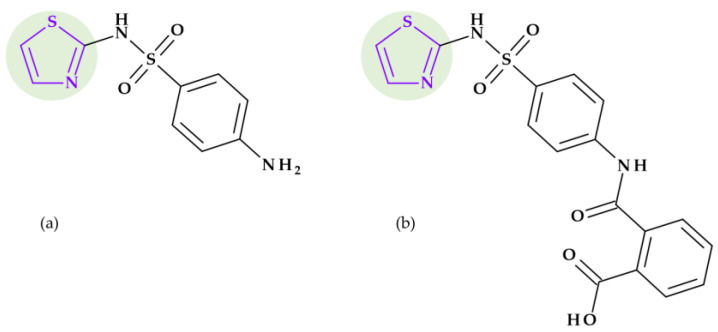
The thiazole heterocycle (purple) depiction in (**a**) Sulfathiazole and (**b**) Phthalylsulfathiazole molecular structure.

*Sulfathiazole.* Sulphathiazole, 4-amino-N-(1,3-thiazol-2-yl)benzenesulfonamide, includs a 1,3-thiazole heterocycle (Figure 31a). Further, its derivatives, including succinylsulphathiazole monohydrate and phthalylsulphathiazole, have been developed [181]. Sulfathiazole is a sulfonamide with a short half-life (8 h) and characteristics comparable to sulfamethoxazole (Section 6.2.3). Due to its toxicity, it is rarely systemically utilized [29,123]. Later, sulfadiazine replaced sulfathiazole due to its broad antibacterial range, potent in vivo activity, low toxicity, and relatively long duration of action [182]. Thus, sulfathiazole is used with other medications to treat skin infections. It is used in formulations for the topical treatment of vaginal infections. For treating ocular infections, sulfathiazole sodium has been topically administered with other medications [123].

*Phthalylsulfathiazole.* Phthalylsulfathiazole, 2-[[4-(1,3-thiazol-2-ylsulfamoyl)phenyl]carbamoyl] benzoic acid, is an N4-acylated sulfonamide (Figure 31b). It is considered a prodrug of sulfathiazole containing a phthalic acid residue at the N4 position [183]. Sulfathiazole is released from phthalylsulfathiazole by bacterial enzymes in the colon through amide hydrolysis [31]. Only around 5% of phthalylsulfathiazole is slowly hydrolyzed to sulfathiazole and is absorbed, leaving about 95% in the colon. It is administered with other antibacterials to treat gastrointestinal tract infections and clean the bowels before surgery [123,183].

### 8.3. Thiadiazoles

Thiadiazoles are five-membered, aromatic, weakly basic, planar, electron-deficient heterocyclic ring systems composed of two carbon atoms, one sulfur atom, and two nitrogen atoms (Table 2). Four non-interconvertible regioisomeric configurations of thiadiazole are achievable: 1,2,3-, 1,2,4-, 1,2,5-, and 1,3,4-thiadiazole [24,184,185]. Among them, 1,2,4-thiadiazoles and 1,3,4-thiadiazoles are found in the structure of some antibacterial compounds.

- *1,2,4-Thiadiazoles.* A five-membered, unsaturated, conjugated heteroaromatic with one sulfur atom and two nitrogen atoms, one of which is next to the sulfur and the other of which is one carbon distinct from it, is known as 1,2,4-thiadiazole. It is a π-excessive ring though relatively π deficient at the two carbon atoms. The nucleophilic substitution is easy at the C5 position due to the lowest π-electron density [24]. In nucleophilic substitution reactions, the C5 position of 1,2,4-thiadiazoles is the most reactive. The 1,2,4-thiadiazoles exhibit extremely few electrophilic reactions. Considering acid-base properties, 1,2,4-thiadiazoles are weak bases. They produce salts with mineral acids, and with heavy metal salts generate additional compounds [186]. Derivatives of 1,2,4-thiadiazoles are useful as antibiotics, cysteine protease inhibitors (cysteine protease cathepsin K), melanocortin-4-receptor agonists, and modulators of adenosine A3 receptors. Also, agrochemicals such as pesticides, soil fungicides, lubricating greases and vulcanization agents are found as derivatives of 1,2,4-thiadiazoles [24,186,187].
- *1,3,4-Thiadiazoles.* 1,3,4-Thiadiazoles are a five-membered, aromatic, weakly basic, planar, electron-deficient heterocyclic ring system composed of two carbon atoms, one sulfur atom, and two nitrogen atoms that resemble pyridine in the N3- and N4-positions of the ring. The 1,3,4-thiadiazole’s dipole moment indicates that it is a polar, symmetric molecule with pseudo-aromatic properties. Due to the inductive influence of nitrogen and sulfur, the carbon atoms at the C2- and C5-positions are electron deficient, so they’re inert to electrophilic substitution but reactive to nucleophilic attack. The high aromaticity of the ring and the inductive action of sulfur are responsible for the 1,3,4-thiadiazole’s weak basicity. In aqueous acidic media, it is relatively stable. However, in an aqueous base, it does not undergo ring cleavage [24]. Numerous medications, including antibiotic, anti-inflammatory, anti-hypertensive, anti-HIV, anti-depressant, local anesthetic, and anti-convulsant drugs, have been discovered based on the 1,3,4-thiadiazole ring [24,29,184].

#### 8.3.1. Beta-Lactam Antibiotics

**Cefalosporins**

A 1,2,4-thiadiazole ring is included in the chemical structure of three fourth-generation cephalosporins: cefozopran, ceftaroline fosamil, and ceftobiprole. The 1,2,4-thiadiazole moiety has been added to modulate the pharmacokinetic characteristics of these antibiotics [186]. In addition, the 1,2,4-thiadiazole enables these cephalosporins to penetrate Gram-negative bacteria and promote transpeptidase action [188,189].

**Figure 32:**
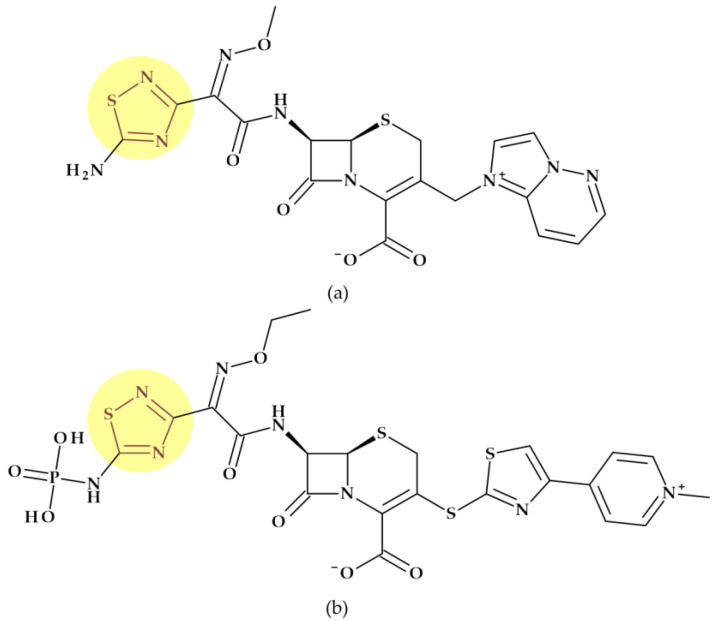
Cephalosporins including thiadiazole heterocycle in their molecular structures: (**a**) Cefozopran, and (**b**) Ceftaroline fosamil.

*Cefozopran* (4th generation). Cefozopran, (6*R*,7*R*)-7-[[(2Z)-2-(5-amino-1,2,4-thiadiazol-3-yl)-2-methoxyiminoacetyl]amino]-3-(imidazo [1,2-b]pyridazin-1-ium-1-ylmethyl)-8-oxo-5-thia-1-azabicyclo [4.2.0]oct-2-ene-2-carboxylate, is a parenteral fourth-generation cephalosporin used as the hydrochloride salt in Japan since the late (Figure 32a) 1990s [123,190,191]. Cefozopran is characterized by a broad spectrum activity against Gram-positive and Gram-negative bacteria. Also, cefozopran exhibits potent activity against Enterococci and *Pseudomonas aeruginosa*, two bacteria resistant to other cephalosporins. Clinically, cefozopran has been approved for the parenteral treatment of various adult patients, including pneumonia, sepsis, urinary tract infections, and intra-abdominal infections [191].

*Ceftaroline fosamil* (5th generation). Ceftaroline fosamil, (6*R*,7*R*)-7-[[(2*Z*)-2-ethoxyimino-2-[5-(phosphonoamino)-1,2,4-thiadiazol-3-yl]acetyl]amino]-3-[[4-(1-methylpyridin-1-ium-4-yl)-1,3-thiazol-2-yl]sulfanyl]-8-oxo-5-thia-1-azabicyclo [4.2.0]oct-2-ene-2-carboxylate, is a cephalosporin antibiotic from the fifth-generation (Figure 32b) [192,193]. The prodrug ceftaroline fosamil is transformed in vivo into ceftaroline, the active metabolite. An additional phosphonic group in the chemical structure of ceftaroline fosamil is the component that confers its name. The advantage of the resulting prodrug is the increased water solubility [188]. Based on the structure of fourth-generation cefozopran, ceftaroline is an oxyimino compound [188,193]. The enhanced action against MRSA is achieved by the existence of the oxime group in the C7 acyl moiety and a 1,3-thiazole ring linked to the central ore (C3 position) [188]. Both common Gram-negative (G(-)) and Gram-positive (G(+)) pathogens, such as MRSA and *Streptococcus pneumoniae*, are susceptible to ceftaroline fosamil [188,193]. Ceftaroline fosamil has received approval to treat ABSSSI and CABP [194].

*Ceftobiprole* (4th generation). Ceftobiprole was briefly discussed in Section 4.1.1 regarding beta-lactam antibiotics, including pyrrole in their molecular structure (Figure 3a). Its C7-side chain contains a 5-amino-1,2,4-thiadiazole heterocycle. 

A representative from the class of cephalosporins includes in its chemical structure a 1,3,4-thiadiazole ring. 

*Cefazolin* (1st generation). This first-generation cephalosporin was previously briefly discussed in Section 4.5.1. In addition to the tetrazole heterocycle from the C7-side-chain, cefazolin contains a [(5-methyl-1,3,4-thiadiazol-2-yl)sulfanylmethyl] fragment at C3 position (Figure 14a) that resembles vitamin-K antagonists in certain respects [195]. To obtain this cephalosporin, this structural fragment has replaced the C3 acetoxy function, including a 1,3,4-thiadiazole heterocycle [29]. Among the adverse reactions of cefazolin that have been reported are coagulation issues linked to hypovitaminosis K. These effects would be exacerbated in impaired renal function when high doses are administered, resulting in the accumulation of its active metabolite. According to some researchers, certain cefazolin-treated patients may benefit from systematic vitamin-K therapy [195].

#### 8.3.2. Sulfonamides

Furthermore, one member of the sulphonamide class has a 1,3,4-thiadiazole ring in its chemical structure. 

**Figure 33:**
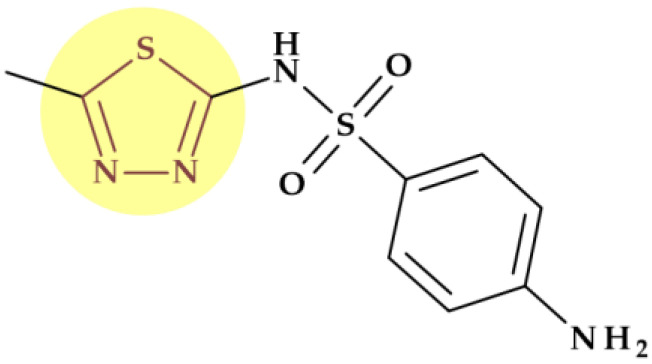
Sulfamethizole molecular structure highlighting 1,3,4-thiadiazole ring.

*Sulfamethizole.* Sulfamethizole is known as 4-amino-N-(5-methyl-1,3,4-thiadiazol-2-yl)benzenesulfonamide (Figure 33) [29]. Approximately 90% of sulfamethizole has been bound to plasma proteins and is easily absorbed from the gastrointestinal system. The half-life of this sulfonamide is between 1.5 and 3 h (a short-acting sulfonamide). Sulfamethizole was orally used to treat urinary tract infections, often combined with other antibacterials. However, because the drug only reaches relatively low concentrations in the blood and tissues, it is ineffective in treating systemic infections [123].

## 9. Conclusions

The role of five-membered heterocycles in the molecular structure of antibacterial drugs used in therapy is a highly relevant and promising area of research in medicinal chemistry and pharmaceutical sciences. Designing antibacterial compounds heavily relies on heterocycles. Incorporating five-membered heterocycles into drug molecules holds promise for improving the antibacterial activity of these drugs. These heterocyclic rings introduce critical structural elements that enable interactions with specific bacterial targets, potentially increasing the efficacy of antibacterial therapies. We have identified the classes of antibacterials of which some representatives include in their molecular structure one or more five-membered heterocycles such as beta-lactam antibiotics, beta-lactamase inhibitors, antibacterial fluoroquinolones, lincosamides, macrolides, nitroimidazoles, oxazolidinones, streptogramins, sulfonamides, and tetracyclines.

The five-membered heterocycles used in antibacterial design contain one to four heteroatoms such as nitrogen, oxygen, or sulfur atoms. We identified in the structure of antibacterials five-membered heterocycles containing:

- One or more nitrogen atoms: pyrrolidine, imidazole, 2-imidazolidinone, 1,2,3-triazole, and tetrazole;
- One oxygen atom: furan;
- Oxygen and nitrogen atoms: 1,3-oxazolidine, oxazole, and isoxazole;
- One sulfur atom: thiophene;
- One sulfur and nitrogen atoms: 1,3-thiazolidine, 1,3-thiazole, and thiadiazoles.

The physicochemical properties of a five-membered heterocycle can play an essential role in determining the biological activity of an antibacterial drug. These properties can affect the antibacterials’ spectrum of activity and potency and their pharmacokinetic, pharmacologic, and toxicological properties. The size and shape of the heterocycle can influence its interaction with target bacterial enzymes, leading to different potency levels against specific bacteria. The presence of certain functional groups on the heterocycle can also impact its ability to penetrate through bacterial cell membranes, affecting its spectrum of activity. 

Additionally, the physicochemical properties of the heterocycle can affect the antibacterials’ pharmacokinetic characteristics. For example, pyrrolidine moiety increased the spectrum of activity against Gram-positive bacteria, including MRSA, and improved pharmacokinetic profile with increased half-life and bioavailability of fluoroquinolones. Triazole enhanced a compound’s solubility and pharmacokinetic and pharmacodynamic features by creating hydrogen bonds (dipole-dipole interactions). Tetrazole acted as a bioisoster of natural amino acids and carboxylic acids, enhancing the pharmacokinetic profile of drugs with therapeutic efficacy. It lowered polarity and raised lipophilicity for improved membrane permeability. An oxazole heterocycle decreases the toxicity of a compound. The 1,2,4-thiadiazole addition to some cephalosporins, such as cefozopran and ceftaroline fosamil, modulates their pharmacokinetic characteristics. Furthermore, the physicochemical properties of the heterocycle can also influence the drug’s pharmacologic and toxicological properties. Certain chemical features may contribute to specific biological effects, such as the inhibition of bacterial enzymes or disruption of essential cellular processes in bacteria. 

Particular heterocycles can affect the drug’s potential toxicity. Several examples are the pyrrolidine substituent in the C7 position of several fluoroquinolones associated with multiple side effects, tetrazolyl acetylamino moiety at the C7 position in some cephalosporins associated with a higher prevalence of hypoprothrombinemia, and pyridine-imidazole group in the telithromycin side chain associated with severe side effects (e.g. hepatotoxic effects).

In summary, an antibacterial with enhanced biological activity, pharmacokinetic profiles, and safety can be designed by comprehending and improving the physicochemical characteristics of the five-membered heterocycles. Incorporating five-membered heterocycles into the molecular structure of antibacterial drugs represents a promising avenue for advancing antibacterial drug development. It offers opportunities to enhance drug efficacy, safety, and diversity, which is crucial in the ongoing battle against bacterial infections and antibiotic resistance. Continued research in this area is essential to translating these promising findings into effective treatments that can benefit global public health.

## Figures and Tables

**Table 1:** FDA-approved antibiotics and their essential heterocycles during 1980–2023 [18,19].

FDA Approval Year	Antibiotic Compound	Antibiotic Class (Generation)	Five-Member Heterocycle in the Structure
1980	Cefotaxime	Beta-lactam cephalosporin (2nd generation)	1,3-Thiazole
1981	Cefoperazone	Beta-lactam cephalosporin (3rd generation)	Tetrazole
1981	Cefotiam	Beta-lactam cephalosporin (2nd generation)	1,3-Thiazole, Tetrazole
1982	Ceftriaxone	Beta-lactam cephalosporin (3rd generation)	1,3-Thiazole
1982	Latamoxef/Moxalactam	Beta-lactam, oxacephem cephalosporin (1st generation)	Tetrazole
1983	Cefonicid	Beta-lactam cephalosporin (2nd generation)	Tetrazole
1983	Cefuroxime	Beta-lactam cephalosporin (2nd generation)	Furan
1984	Ceftazidime	Beta-lactam cephalosporin (2nd generation	1,3-Thiazole
1986	Aztreonam	Beta-lactam monobactam	1,3-Thiazole
1987	Cefotetan	Beta-lactam cephalosporin (3rd generation)	Tetrazole
1992	Cefpodoxime proxetil	Beta-lactam cephalosporin (3rd generation)	1,3-Thiazole
1991	Cefuroxime axetil	Beta-lactam cephalosporin (3rd generation)	Furan
1992	Ceftibuten	Beta-lactam cephalosporin (3rd generation)	1,3-Thiazole
1992	Tazobactam	Beta-lactamase inhibitor	1,2,3-Triazole
1993	Cefixime	Beta-lactam cephalosporin (3rd generation)	1,3-Thiazole
1996	Cefepime	Beta-lactam cephalosporin (4th generation)	Pyrrolidine, 1,3-Thiazole
1996	Meropenem	Beta-lactam carbapenem	Pyrrolidine
1997	Cefdinir	Beta-lactam cephalosporin (4th generation)	1,3-Thiazole
2000	Linezolid	Oxazolidinone	1,3-Oxazolidine
2001	Ertapenem	Beta-lactam carbapenem	Pyrrolidine
2001	Telithromycin	Ketolide macrolide	Imidazole
2003	Gemifloxacin	Fluoroquinolone	Pyrrolidine
2009	Ceftobiprole	Beta-lactam cephalosporin (5th generation)	Pyrrolidine
2014	Doripenem	Beta-lactam carbapenem	Pyrrolidine
2014	Finafloxacin	Fluoroquinolone	Pyrrole (in a bicycle)
2014	Tedizolid	Oxazolidinone	1,3-Oxazolidin-2-one, Tetrazole
2018	Eravacycline	Tetracycline	Pyrrolidine
2018	Gemifloxacin	Fluoroquinolone	Pyrrolidine
2019	Imipenem + Cilastatin + Relebactam	Relebactam: beta-lactamase inhibitor	2-Imidazolidinone (in an azabicycle)
2019	Cefidorocol	Beta-lactam cephalosporin (5th generation)	Pyrrolidine, thiazole
2021	Ceftidoren pivoxil	Beta-lactam cephalosporin (5th generation)	Thiazole (2 groups)
2023	Sulbactam + durlobactam	Sulbactam: beta-lactam antibacterial and beta-lactamase inhibitor Durlobactam: beta-lactamase inhibitor	Sulbactam: 1,3-Thiazolidine 1,1-dioxide Durlobactam: 2-Imidazolidinone (in an azabicycle)

**Table 2:** The most common heterocycles with five atoms found in the molecular structure of antibacterial agents (HBA—Hydrogen Bond Acceptor Count, HBD—Hydrogen Bond Donor Count, MW—Molecular Weight) [21].

Five-Membered Heterocycles	Heteroatom (s)	Chemical Structure	MW (g/mol)	HBA	HBD
Pyrrolidine	N	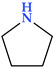	71.12	1	1
Imidazole	N(2)	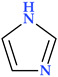	68.08	1	1
1,2,3-Triazole	N(3)	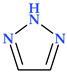	69.07	2	1
Tetrazole	N(4)	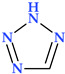	70.05	3	1
Furan	O(1)	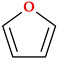	68.07	1	0
1,3-Oxazolidine	N(1),O(1)	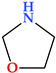	73.09	2	1
1,3-Oxazole	N(1),O(1)	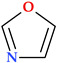	69.06	2	0
1,2-Oxazole (Isoxazole)	N(1),O(1)	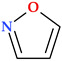	69.06	2	0
Thiophene	S(1)	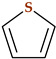	84.14	1	0
1,3-Thiazolidine	N(1),S(1)	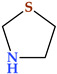	89.6	2	1
1,3-Thiazole	N(1),S(1)	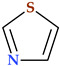	89.16	2	1
1,2,4-Thiadiazole	N(2),S(1)	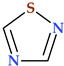	86.12	3	0
1,3,4-Thiadiazole	N(2),S(1)	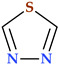	86.12	3	0

**Table 3:** Groups of antibiotics with a pyrrolidine moiety in the chemical structures (Ref. = References).

No.	Therapeutic Class	Subclass	Reprezentatives	Ref.
1	Beta-lactam antibiotics	Carbapenemes	Doripenem	[30]
			Ertapenem	[30]
			Meropenem	[30]
		Cephalosporins	Cefepime	[31]
			Cefiderocol	[32]
			Ceftobiprole	[33]
2	Fluoroquinolones	-	Clinafloxacin	[34]
			Finafloxacin	[34]
			Gemifloxacin	[34]
			Lascufloxacin	[34]
			Premafloxacin	[35]
			Sitafloxacin	[34]
			Trovafloxacin	[34]
3	Lincosamides	-	Lincomycin	[31]
			Clindamycin	[31]
4	Streptogramins	-	Quinupristin/Dalfopristin	[36]
5	Tetracyclines	-	Rolitetracycline	[29]
		Glycylcyclines	Eravacycline	[37]

**Table 4:** Associated side-effects of pyrrolidine moiety in comparison with other possible substituents in the C7 position of fluoroquinolones [54].

Associated Side-Effects	Comparison with Other Substituents in the C7 Position
Genotoxicity	Pyrrolidine > Piperazine > Alkyl
	Pyrrolidine (unsubstituted) > Piperazine (unsubstituted) > Pyrrolidine (substituted) > Piperazine (substituted)
Neuropsychiatric toxicity, seizures (GABA receptor binding)	Alkyl > Piperazine (unsubstituted) > Pyrrolidine (unsubstituted) > Piperazine (substituted) or Pyrrolidine (substituted)
Some NSAIDs interactions	Piperazine (unsubstituted) > Pyrrolidine (unsubstituted) > Piperazine (substituted) or Pyrrolidine (substituted)
Theophylline interactions	Pyrrolidine (unsubstituted) > Piperazine (unsubstituted) > Piperazine (substituted) or Pyrrolidine (substituted)

**Table 5:** Cephalosporins containing a 2-amin-1,3 thiazole heterocycle in the 7-acyl side chain [21,29,123,176].

No.	Cephalosporin	Generation	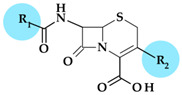		Administration	t_1/2_ (Hours)	Acid Resistant	Resistance to β-lactamases	Antibacterial Spectrum	Activity against *Pseudomonas* sp.
			R1	R2						
1	Cefpodoxime (proxetil)	3rd	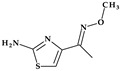	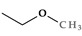	Oral	2.2	Yes	Good	Extended	No
2	Cefotaxime	3rd	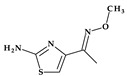	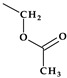	Parenteral	1	No	Good	Extended	Yes
3	Ceftazidime	3rd	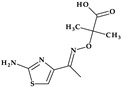	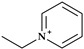	Parenteral	2	No	Good	Extended	Yes
4	Ceftriaxone	3rd	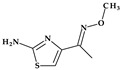	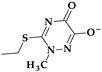	Parenteral	6–9	No	Good	Extended	Yes
5	Cefixime	3rd	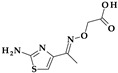	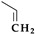	Oral	3–4	Yes	Good	Extended	No
6	Cefdinir	3rd	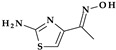	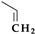	Oral	1.7	Yes	Good	Extended	No
7	Cefditoren (pivoxil)	3rd	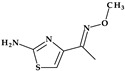	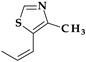	Oral	1.6	Yes	Good	Extended	No
8	Ceftibuten	3rd	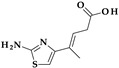	-H	Oral	2–2.3	Yes	Good	Extended	No
9	Ceftizoxime	3rd	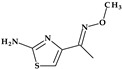	-H	Parenteral	1.7	No	Good	Extended	Yes
10	Cefepime	4th	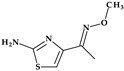	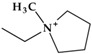	Parenteral	2	No	Good	Extended	Yes
11	Cefpirome	4th	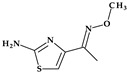	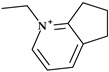	Parenteral	2	No	Good	Extended	Yes
